# Leveraging transcriptomics-based approaches to enhance genomic prediction: integrating SNPs and gene networks for cotton fibre quality improvement

**DOI:** 10.3389/fpls.2024.1420837

**Published:** 2024-09-20

**Authors:** Nima Khalilisamani, Zitong Li, Filomena A. Pettolino, Philippe Moncuquet, Antonio Reverter, Colleen P. MacMillan

**Affiliations:** ^1^ Cotton Biotechnology, Agriculture and Food, CSIRO, Canberra, ACT, Australia; ^2^ Livestock and Aquatic Genomics, Agriculture and Food, CSIRO, St Lucia, QLD, Australia

**Keywords:** cotton, gene-networks, fibre quality, cell-wall, genomic prediction, breeding

## Abstract

Cultivated cotton plants are the world’s largest source of natural fibre, where yield and quality are key traits for this renewable and biodegradable commodity. The *Gossypium hirsutum* cotton genome contains ~80K protein-coding genes, making precision breeding of complex traits a challenge. This study tested approaches to improving the genomic prediction (GP) accuracy of valuable cotton fibre traits to help accelerate precision breeding. With a biology-informed basis, a novel approach was tested for improving GP for key cotton fibre traits with transcriptomics of key time points during fibre development, namely, fibre cells undergoing primary, transition, and secondary wall development. Three test approaches included weighting of SNPs in DE genes overall, in target DE gene lists informed by gene annotation, and in a novel approach of gene co-expression network (GCN) clusters created with partial correlation and information theory (PCIT) as the prior information in GP models. The GCN clusters were nucleated with known genes for fibre biomechanics, i.e., fasciclin-like arabinogalactan proteins, and cluster size effects were evaluated. The most promising improvements in GP accuracy were achieved by using GCN clusters for cotton fibre elongation by 4.6%, and strength by 4.7%, where cluster sizes of two and three neighbours proved most effective. Furthermore, the improvements in GP were due to only a small number of SNPs, in the order of 30 per trait using the GCN cluster approach. Non-trait-specific biological time points, and genes, were found to have neutral effects, or even reduced GP accuracy for certain traits. As the GCN clusters were generated based on known genes for fibre biomechanics, additional candidate genes were identified for fibre elongation and strength. These results demonstrate that GCN clusters make a specific and unique contribution in improving the GP of cotton fibre traits. The findings also indicate that there is room for incorporating biology-based GCNs into GP models of genomic selection pipelines for cotton breeding to help improve precision breeding of target traits. The PCIT-GCN cluster approach may also hold potential application in other crops and trees for enhancing breeding of complex traits.

## Introduction

Cotton (*Gossypium* spp.) is one of the most economically significant crops, providing fibre and oilseed products worldwide. In recent years, there has been an increasing demand for cotton with improved agronomic traits, including for textile quality. Traditional phenotype-based cotton breeding approaches have been limited by the time-consuming and labour-intensive nature of phenotypic evaluation, significant costs, and the underlying genetic complexity of major traits such as yield and quality traits. For example, a cotton variety typically takes 10 years of traditional breeding, and large numbers of plants and field sites. Furthermore, the cotton genome is large and very complex as it is an allotetraploid with a long evolutionary lineage of divergence and domestication spanning before that of hexaploid wheat and tetraploid canola (Li et al., 2015; Chen et al., 2020). These issues make breeding of complex multi-genic traits difficult and slow. To help accelerate breeding, genomic prediction (GP) is a relatively recent strategy being researched in cotton (Conaty et al., 2022) and also in breeding of a wide range of crops and trees that utilise genome-wide data to estimate breeding values. GP enables genomic selection (GS) of valuable germplasm with desirable traits early in the breeding process based on genetic information, and consequently helps to significantly shorten the breeding cycle, reduces resource intensity associated with large amounts of phenotyping, and facilitates acceleration of genetic gain and precision of target traits (Budhlakoti et al., 2022). Furthermore, advancements in high-throughput sequencing technologies have raised many possibilities for the integration of omics-based approaches, particularly transcriptomics, to help accelerate the potential of GS and GP strategies in cotton breeding for accelerating genetic gain for a range of significant phenotypes such as fibre quality traits of elongation, strength, length, disease resistance against verticillium and fusarium wilts and other fungi as well as insect pests such as silver-leaf white fly, and water and sodium stress tolerance (Conaty et al., 2022).

Cotton seed fibre quality traits are important considerations in the global commodity market. These economically important traits include fibre length, strength, and elongation, among others, such as micronaire, short fibre index, uniformity, and colour. A key component of fibre quality is the cell wall whose composition plays a pivotal role in determining the fibre’s physical properties (Pettolino et al., 2022). Although thousands of genes are expressed during fibre development and some important genes were identified (MacMillan et al., 2017; Wang et al., 2017; Ma et al., 2018; Li et al., 2020; Gallagher et al., 2020; Li et al., 2021a; Yang et al., 2023), the majority of all the contributors to fibre traits remain largely unidentified. Known cell wall genes that affect the strength and elongation properties of the cell walls of a range of angiosperm fibres include fasciclin-like arabinogalactan proteins (FLAs) (MacMillan et al., 2010, 2015; Ma et al., 2022), as well as cellulose synthases and cell wall biosynthetic enzymes that affect fibre quality traits such as length, strength, and elongation (Liang et al., 2015; Huang et al., 2015). Understanding the regulatory mechanisms of and the interaction among these genes is crucial for unravelling the genetic basis of these desirable traits.

Transcriptomics allows for the quantification of gene expression levels across different tissues, developmental stages, and environmental conditions. By utilising differential expression (DE) analysis, many researchers have identified the possibility of identifying genes that are differentially regulated in response to favourable traits, thus using a biological basis for precision breeding approaches. This information, in turn, has been flagged as useful to guide the development of precise breeding strategies using biological understanding. Transcription-wide association studies is a relatively new approach that is being explored in studies for tackling human diseases (Mai et al., 2023) and in different plant species to determine accurate gene-level association mapping (Li et al., 2021a). Moreover, transcriptomic data have been providing valuable insights into the molecular mechanisms underlying these traits, aiding in the discovery of candidate genes and potential regulatory networks involved in their development (Zhang et al., 2015). In cotton, transcriptomics studies have identified large numbers of genes associated with a range of fibre traits by using genome-wide association studies (GWAS) and QTL approaches identifying genes for strength and maturity in cotton fibres (Wang et al., 2017; Ma et al., 2018; Li et al., 2021b).

Gene network analysis further enhances our understanding of the complex interactions between genes, proteins, and regulatory elements, and how they contribute to the expression of desirable traits in cotton. By constructing gene co-expression networks (GCNs), it is now possible to identify highly interconnected gene networks that may be functionally related and potentially associated with the phenotypic traits of interest, using computational graphical learning approaches such as those with their origin based on partial correlation and information theory (PCIT) (Reverter and Chan, 2008; Watson-Haigh et al., 2009). Groups of genes as clusters within these networks can serve as key regulators or biomarkers of the traits of interest, offering opportunities for targeted manipulation or marker-assisted selection (Xu et al., 2011; Deng et al., 2012; Gu et al., 2020; Zhang et al., 2022).

Integrating omics-based data, in particular the transcriptome data, with GP approaches is becoming an increasingly popular strategy considered to hold potential for enhancing plant breeding including for cotton (e.g., Hu et al., 2019; Azodi et al., 2020; Liu et al., 2020, 2022; Wang et al., 2023; Yang et al., 2023). Furthermore, single-nucleotide polymorphisms (SNPs) are commonly used as molecular markers in GS, capturing the genetic variation responsible for phenotypic variation. By incorporating transcriptomic information, which is more directly linked to phenotypes than SNPs, there could be potential to increase the accuracy of GP (Hu et al., 2019). One strategy for this is to measure the transcriptome data for the lines available for the GP study and then integrate the transcriptome and SNP data to jointly conduct prediction analysis (e.g., Hu et al., 2019; Perez et al., 2022). Alternatively, DE analysis can be conducted using transcriptome data alone, and then the DE genes detected from the analysis can be used as knowledge in the GP model, e.g., by adding a specific weight to the effects of SNPs that are linked to the DE regions. The potential value of weighting genes in co-expression networks has been flagged for cotton breeding in a study of exploring gene function of fibre development (Zhang et al., 2022). Innovative approaches taken in this research outlined here include using not only DE genes, but also outputs from gene network analysis based on PCIT, as well as inclusion of biologically relevant developmental time points.

**Figure 1:**
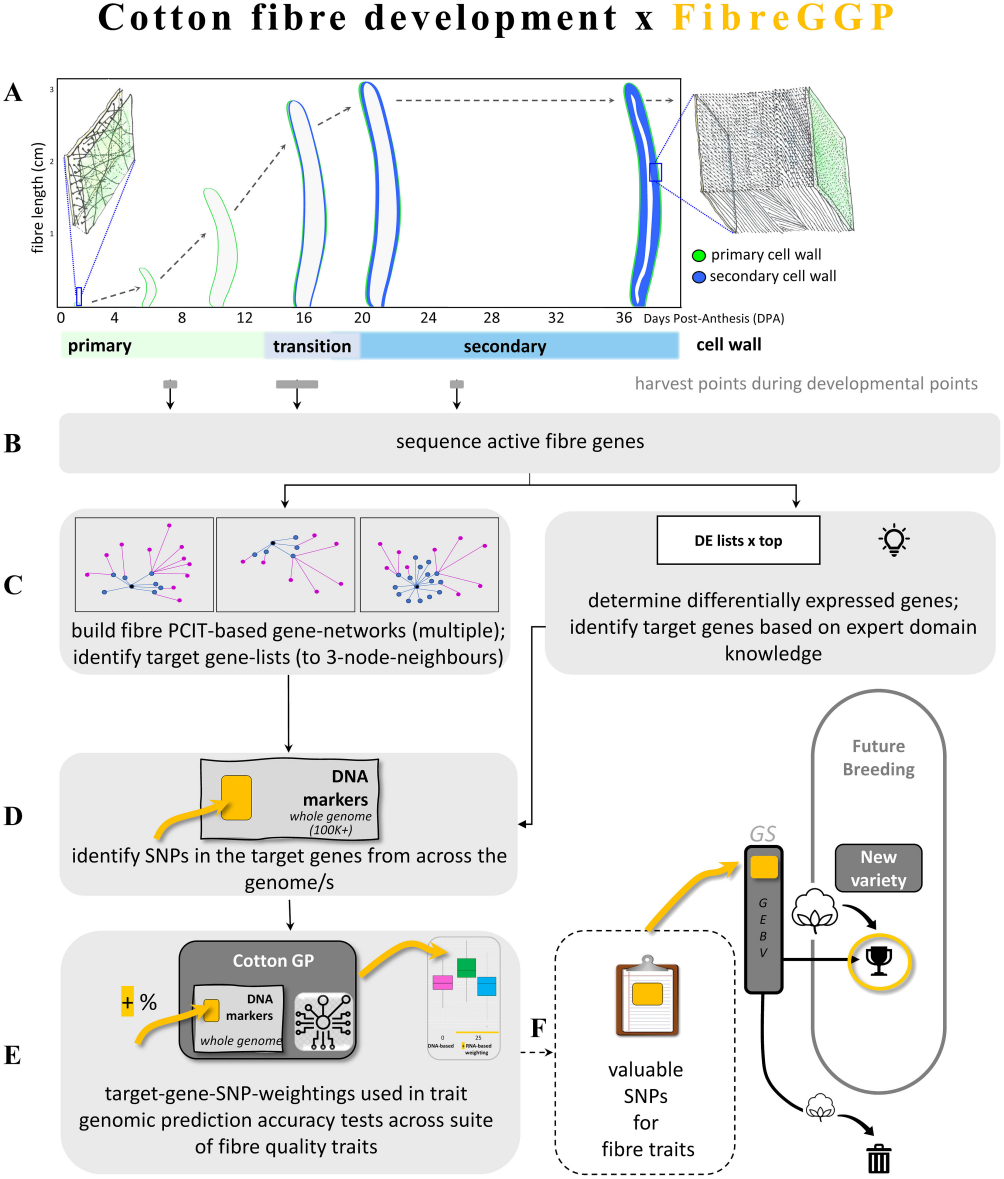
Cotton fibre development and the FibreGGP workflow undertaken in this study, and its links to cotton breeding. **(A)** Fibre genes expressed in key points of development are identified through targeted harvesting and RNA sequencing. A fibre cell emerges on the surface of the cotton seed (ovules) on the day post anthesis (flowering; i.e., 0 DPA); this fibre cell has a thin expanding primary cell wall. The fibre cell grows to several centimetres long over a period of 2–3 weeks. Towards the end of its growth phase, the fibre cell wall starts its transition to deposition of the secondary wall that continues for several weeks until a very thick secondary wall has been formed, and the cell matures, dehydrates, and dies. The mature fibre is typically harvested for commercial purposes once the cotton boll has fully opened ~60+ DPA. Fibre-expressed genes were obtained at three points in development (shown in grey bars), i.e., primary growth phase at 7 DPA, a transition point at an average of 16 DPA, and during secondary cell wall formation once fibre cell growth had largely ceased at 25 DPA. **(B)** The fibre-expressed genes are sequenced via RNA-seq. **(C)** Fibre-expressed genes are analysed in two ways to create target gene lists, via “PCIT gene networks”, i.e., that generate specialist gene networks using PCIT and up to three node neighbours in key gene networks, and via “DE lists”, i.e., differential gene-expression top lists via expert domain knowledge. **(D)** The target gene list networks guide the identification of SNPs based on the expressed fibre genes. **(E)** The fibre-guided SNPs are weighted in GS algorithms and tested for improved prediction accuracy of key fibre traits. **(F)** How the target SNPs are integrated into the breeding pipeline (dashed line; not part of this study). Long and short lists of desirable target SNPs yielding positive results are potentially fed into the cotton breeding pipeline. GEBV, Genetic Estimated Breeding Values. FibreGGP, Fibre Gene-network guided Genomic Prediction.

Biologically relevant transcriptomes can be important for identifying the DNA elements involved in a trait. For this study, a key element is the capture of synchronous single-cell transcriptomes across biologically relevant developmental stages (**Figure 1A**). The cotton seed fibre is a single cell, and its development follows a clear sequence, from seed fibre cell initiation just before the day of flowering, rapid cell growth to >3 cm long with a soft primary cell wall (PCW) over ~2.5 weeks, a transition phase, a secondary cell wall (SCW) deposition phase during which cell extension ceases and a thick secondary cell wall is deposited on the inner surface of the cell’s primary wall, a cell maturation and programmed cell death phase, and then finally yielding a dry mature fibre cell after ~2 months that is harvested for trade on the global natural fibre commodity market.

In this study, we aimed to investigate the potential of integrating transcriptomic information obtained through RNA-seq analysis as a pre-knowledge in GP models in cotton breeding. We determined whether targeted enrichment of SNPs from groups of genes that affect a fibre quality trait could provide better GP than random SNPs. Such an approach would identify subsets of specific genes from across the large suite of ~80K genes in the cotton genome that contribute to the formation of a desired trait, thus taking a biology-informed approach to GP where RNA expression is used in GP as compared to only DNA markers. This would hold benefit to zoom in to the specific genes for a trait, thus accelerating the precision of GP. By leveraging developmental time course DE and gene network analysis, we identified candidate genes associated with fibre length, elongation, and strength. Next, these genes were considered as prior information in a well-known Bayesian GP model (i.e., Bayes C model), where a small number of SNPs located within or near the relevant gene regions were assigned with a different prior, which weighted their genetic effects more heavily than others. We then evaluated whether this weighted Bayesian model provided enhanced prediction accuracy compared to the standard Bayes C model, on a previously published cotton genome selection data set collected from the CSIRO breeding program (Li Z. et al., 2022). This research has the potential to advance cotton breeding strategies by providing a more comprehensive understanding of the genetic architecture underlying desirable traits and enabling the identification of superior genotypes at an earlier stage. This could extend to additional fibre traits such as yield, additional quality traits such as micronaire, maturity, uniformity, short fibre index, and colour as well as disease resistance traits such as against fungal wilts and against insect/arthropod pests.

The foundation of the workflow undertaken in this study is the biology of the cotton seed fibre. This framework—”Fibre Gene-network Guided Genomic Prediction” (FibreGGP)—is outlined in **Figure 1**. Gene expression at specific fibre developmental points was determined with RNA extracted at key points in the primary, transition, and secondary cell wall stages of the cell’s development (**Figure 1A**). In this study, we interrogate the expression of all the genes expressed at each of these developmental points (**Figure 1B**). To examine potential improvements in GP accuracy of cotton fibre quality traits, a Bayesian regression approach using various summary statistics derived from transcriptome data analysis as prior information was tested in targeted genes from the cotton genome (**Figures 1C–E**) across three scenarios. Scenario 1 tested all the DE genes arising from pairwise comparisons. Scenario 2 tested select DE genes that were considered biologically relevant to the trait of interest. Scenario 3 tested a highly targeted set of DE genes derived from PCIT-based GCNs based on particular key genes, and their first, second, and third network neighbours. In this scenario, we tested key genes linked to the biomechanical properties of strength and elasticity of angiosperm fibre secondary cell walls, i.e., a sub-class of FLAs. Ultimately, our framework’s aim is to enhance the precision breeding of cotton for select traits (**Figure 1F**).

## Materials and methods

### Transcriptomics, genomics, and phenomics data

The transcriptome data used in this study were retrieved from MacMillan et al. (2017). Briefly, the authors grew *G. hirsutum* Coker 315-C11 plants in natural summer light in a glasshouse at 31°C (day; 16 h) and 26°C (night; 8 h) in Canberra, Australia. The RNA-seq experiment was designed to capture fibre gene expression at key fibre developmental time points, i.e., at 7 days post-anthesis (DPA) (PCW stage), 14, 15, and 16 DPA (labelled here as “16 DPA”; transition to SCW stage), and 25 DPA (SCW stage) (see **Figure 1**). Three biological replicates as different plants were harvested. Fibre was hand-separated from the seed of each boll under liquid nitrogen, then ground to a fine powder before RNA extraction (Qiagen) and sequencing via Illumina HiSeq2000 to generate the raw transcriptome data (see exact details in MacMillan et al., 2017). Genomic and phenotypic data were obtained from Li Z. et al. (2022), representing 1,907 samples collected from 1994 to 2017 and 12,296 informative SNPs. All the samples were collected from the CSIRO cotton breeding program to mimic the real breeding practice. The genetic diversity of the study population has also been investigated in Li Z. et al. (2022), using both pedigree and genomic data (referring to **Supplementary Figures S1**, **S2** in Li Z. et al., 2022). Our focus was six fibre quality traits, i.e., fibre length (upper half-mean length of sample), strength (the breaking point force of a bundle of fibres of a given weight and fineness, g/tex), elongation (fibre bundle extension force up to its breaking point, expressed as a % increase over its original length), micronaire (a measure of air permeability of compressed fibre samples, which is a composite indication of fibre linear density and maturity, unitless), uniformity (the ratio of the mean fibre length to the upper half-mean length, expressed as %), and short fibre index (the proportion by weight of fibre shorter than 12.7 mm).

### RNA-seq, PCIT, and gene network analyses

RNA-seq analysis was conducted on the raw transcriptome data (above). FastQC software (Andrews, 2010; default parameters) was employed to assess the quality of the RNA-seq data. The *Gossypium hirsutum* TM-1 v3.1 (Phytozome Genome ID 578, NCBI taxonomy ID 3635; Sreedasyam et al., 2024) was used as the reference genome and indexed using HISAT2 (Kim et al., 2015; default parameters; version V2.1) to facilitate the sequence alignment. Afterwards, HISAT2 was used to align the RNA-seq files to the reference genome, generating a Sequence Alignment/Map (.sam) file format. The.sam files were converted to Binary Alignment/Map (.bam) files using SAMtools (Kim et al., 2015; default parameters; V1.12) for further analysis. The mapped files were merged using Cuffmerge (Trapnell et al., 2012; default parameters; V2.2.1), and the resulting merged map file was compared to the reference genome using Cuffcompare (Trapnell et al., 2012; default parameters; V2.2.1). Next, Stringtie (Pertea et al., 2015 default parameters; V2.2.0) was used to estimate the abundance of genes/transcripts in the mapped file. This step resulted in the construction of a gene count matrix for DE analysis. Fragments Per Kilobase of transcript per Million mapped reads (FPKM) was used to measure and normalise expression levels in the analyses. Consequently, the DE analysis was performed using the DESeq2 package (Love et al., 2014; default parameters; V1.44.0), with DE genes identified using a false discovery rate (FDR) threshold of 0.001 *p-*value. Overexpressed and underexpressed genes were identified using a log2foldchange threshold of 1.5 to capture transcription factors (TFs) as well as structural and more abundantly expressed genes. The DE analysis was performed for independent pairwise comparison of three stages of fibre, e.g., fibre 07 vs. 16, fibre 07 vs. 25, and fibre 16 vs. 25.

After DE analysis, PCIT (Reverter and Chan, 2008; Watson-Haigh et al., 2009) was used to estimate the pairwise correlation between DE genes. The PCIT algorithm has two distinct steps: (1) first-order partial correlation coefficients are computed through a defined code sequence that determines the strength of the linear relationship between the expressed genes, and (2) the Data Processing Inequality, a theorem in Information Theory, is used to capture significant associations between the expressed genes where the approach determines significance between node pairs post accounting for all other network nodes (Reverter and Chan, 2008; Watson-Haigh et al., 2009; Fernandes et al., 2024). PCIT produced an output table in the form of a correlation matrix among genes, which was used to produce a GCN. Next, GCNs were generated using Cytoscape 3.9.1 using the PCIT correlation matrix as the input table and then the network was analysed with Cytoscape default network analysis settings. The PCIT-based network was used to identify clusters of genes (GCN clusters) potentially linked to specific fibre quality traits. Each GCN cluster was generated by identifying a key fibre-trait gene, and then identifying a cluster containing that gene’s either 1st, 1st, and 2nd, or 1st, 2nd, and 3rd neighbours by using Cytoscape’s function to display neighbours in a list, and then the said list is exported. The various PCIT-based GCN clusters were generated for Scenario 3 (see below) where the central gene of each cluster was the biomechanics-focussed FLA7, FLA11, or FLA12. The criteria used for selecting the key genes central for each GCN cluster were biology-informed. This was based on demonstrated functional biology and provides a knowledge-based entry point into the large GCN to then determine additional genes of unknown/known function involved in the trait, here determined as GCN clusters. We tested known fibre biomechanics genes—SCW FLAs (MacMillan et al., 2010, 2015, 2017; Ma et al., 2022). The criteria for selection network neighbours had no limitation—all 1st, 2nd, and 3rd network neighbours of the central gene were selected for the GCN cluster.

### Identification of SNPs in target genes/gene regions

Various scenarios were used in the identification of SNPs that were subsequently tested for GP accuracy of various traits. SNPs for various traits were identified based on DE gene lists, expert domain knowledge, and via PCIT-based GCNs.

*Scenario 1:* DE genes were obtained via DESeq2 from three fibre developmental stages comparing fibre 07 vs. 16, fibre 07 vs. 25, and fibre 16 vs. 25. Genome coordinates of the DE genes were identified, followed by identification of SNPs within the DE genes.

*Scenario 2:* Annotation, i.e., biological information from the DE gene lists, was used to identify a subset of SNPs associated with length and strength. Expert domain knowledge of fibre quality traits was employed to identify target genes for these traits, e.g., downregulated genes from the fibre 07 vs. 16 DE gene data set that were considered to be either involved in length, or upregulated genes from fibre 07 vs. 25 DE gene set for strength. In addition, within each data set of this scenario, SNPs were identified either in the exact gene region, within a 1-kb region, or within a 10-kb region. This resulted three sets of SNPs for each trait except for length which did not have SNPs in 1 kb of the DE genes. Using this scenario, there were seven sub-scenarios: L0, L1, L3, S0, S1, S2, and S3, where the number 0 represents an unweighted SNP scheme and numbers 1, 2, and 3 indicate the presence of SNPs in the exact gene location, within 1 kb, and within 10 kb of the selected DE genes, respectively. L represents length and S represents strength.

*Scenario 3:* Three sets of DE genes from the fibre 16 vs. 25 DPA were identified as crucial for fibre quality. These genes were chosen based on biological function and they were FLA7, FLA11, and FLA12 (this was based on known genes closely involved in the biomechanics of plant fibres; MacMillan et al., 2010, 2015). Each of these genes was identified in the GCN, and the genes with which they were co-expressed were identified by generating GCN clusters that included the primary target gene’s 1st, 2nd, and 3rd network neighbours. These lists of GCN cluster genes were used to identify relevant SNPs in those loci followed by analysis of the impact on GP accuracy for the following traits: strength, length, and elongation.

### Bayesian GP model accounting for SNPs associated with DE gene regions

In a GP model, we aimed to add more on the effects of a subset of SNPs linked to DE gene regions or their close neighbours in GCN clusters, which can be done by using specific priors for those SNPs. This idea is illustrated on the well-known Bayes C linear regression model (Habier et al., 2011), with its likelihood model form defined as follows:


(1) $$P\left(y│\beta ,\sigma_{e}^{2}\right)=\prod_{i=1}^{n}\frac{1}{\sqrt{2{\text{πσ}}_{e}^{2}}}\text{exp}\left(-\frac{{\left(y_{i}-{\text{β}}_{0}-\sum_{j=1}^{p}x_{ij}\beta_{j}-W\text{α}-Z\text{γ}\right)}^{2}}{2{\text{σ}}_{e}^{2}}\right),$$

where *y_i_* is the phenotype record of the *i*th individual (*i* = 1,…, *n*; *n* is the total number of individuals), *x_ij_* are the genotype value of the individual *i* and SNP *j*, coded as −1, 0, and 1 for genotypes AA, AB, and BB, respectively, *W* and *Z* are the design matrix for the year and experiment, *α* and *γ* are the associated random effects, *β*_0_ is the model intercept, and *e_i_* is the residual error: $e=\left[e_{1},\ldots ,e_{n}\right]\sim N\left(0,{\text{Iσ}}_{e}^{2}\right)\;\left(\text{mutually independent for }i=1,\ldots ,n\right),{\text{ σ}}_{e}^{2}$ is the residual variance, *β_j_* (*j* = 1, …, *p*) is the regression coefficient representing the additive genetic effect of the marker *j*. The genetic effect *β_j_* was assigned with a spike and slab prior (Ishwaran and Rao 2005) to the regression parameters as follows:


(2) $$P\left({\text{β}}_{j}{\text{│γ}}_{j}\right)\propto \left(1-{\text{γ}}_{j}\right){\text{I}}_{\left({\text{β}}_{\text{j}}=0\right)}+{\text{γ}}_{\text{j}}\text{N}\left({\text{β}}_{\text{j}}│0,\sigma_{\text{b}}^{2}\right),$$

where ${\text{γ}}_{j}$ is a binary indicator variable to tell whether the genetic effect of SNP *j* should be non-negligible and follow a normal distribution, or whether the effect is small and assigned with a zero value. In the standard Bayes C model, the indicator variable ${\text{γ}}_{j}$ and the variance component $\sigma_{\text{b}}^{2}$ are further assigned with priors of Bernoulli: $Bern{\text{(γ}}_{j}\text{│π})$ and Inverse chi-squared: $IG\left(\sigma_{\text{b}}^{2}│df,S_{0}\right)$ , respectively. In the Inverse gamma prior $IG\left(\sigma_{\text{b}}^{2}│df,S_{0}\right)$ , the parameters df = 5 and $S_{0}=\text{var}\left(y\right)\times R^{2}\;\times \left(df+2\right)/MSx$ , with *R* ^2 =^ 0.5, assuming that 50% of the phenotype variance is explained by the whole set of SNPs. In the Bernoulli prior $Bern{\text{(γ}}_{j}\text{│π})$ , the parameter π was further assigned with a Beta prior Beta (π|p_0_, π_0_), with p_0_ = 50 and π_0_ = 0.5. The spike and slab prior (13) are often referred to as the Bayes C model (Habier et al., 2011) in the GP literature.

To add more weights on the effects of SNPs linked to DE gene regions, an alternative prior to (2) was specified:


$$\{\begin{array}{c} P\left({\text{β}}_{j}{\text{│γ}}_{j}\right)\propto \left(1-{\text{γ}}_{j}\right){\text{I}}_{\left({\text{β}}_{\text{j}}=0\right)}+{\text{γ}}_{\text{j}}N\left({\text{β}}_{\text{j}}│0,{\text{σ}}_{\text{DEb}}^{2}\right),\;j\in G\\ P\left({\text{β}}_{j}{\text{│γ}}_{j}\right)\propto \left(1-{\text{γ}}_{j}\right){\text{I}}_{\left({\text{β}}_{\text{j}}=0\right)}+{\text{γ}}_{\text{j}}N\left({\text{β}}_{\text{j}}│0,{\text{σ}}_{\text{non}-\text{DEb}}^{2}\right),\;j\notin G \end{array}$$

where *G* represents a specific subset of SNPs linked to a DE gene region or their neighbours in a GCN cluster, separate variances ${\text{σ}}_{\text{DEb}}^{2}$ and ${\text{σ}}_{\text{non}-\text{DEb}}^{2}$ were given to an SNP depending on whether it was presented in *G*. Those variances were then assigned with different hyper-priors $IG\left({\text{σ}}_{\text{DEb}}^{2}│df,S_{\text{DE}0}\right)$ , and $IG\left({\text{σ}}_{\text{non}-\text{DEb}}^{2}│df,S_{\text{non}-\text{DE}0}\right),$ , respectively, where $S_{\text{DE}0}=\text{var}\left(y\right)\times R_{\text{DE}}^{2}\;\times \left(df+2\right)/MSx$ , and $\text{var}\left(y\right)\times R_{\text{non}-\text{DE}}^{2}\;\times \left(df+2\right)/MSx.\;$ the choice of $R_{\text{DE}}^{2}$ and $R_{\text{non}-\text{DE}}^{2}$ determined how much weights were assumed for SNPs within *G*. We used the following three combinations: (i) $R_{\text{DE}}^{2}=0.125$ , $R_{\text{non}-\text{DE}}^{2}$ =0.375 ; (ii) $R_{\text{DE}}^{2}=0.25,R_{\text{non}-\text{DE}}^{2}\;=0.25;\;$ ; and (iii) $R_{\text{DE}}^{2}=0.375$ , $R_{\text{non}-\text{DE}}^{2}=0.125$ . These settings correspond to the SNPs within *G* assumed to explain 25%, 50%, and 75% of the total genetic variance, respectively. SNPs identified in the DE genes were weighted at either low (25%), medium (50%), or high (75%) levels in the GP calculations to test whether their role in the target traits were significant for improving GP accuracy. The improved prediction accuracy would indicate that these SNPs linked to DE genes or GCN clusters play a more important role than other SNPs, since they were weighted more in the Bayesian regression model to predict fibre qualities. Random weightings of loci were also included in the test scenarios, and these are presented in the results.

The Bayesian C algorithm utilises the Markov Chain Monte Carlo (MCMC) method to sample from the joint posterior distribution of the model parameters. MCMC generates a sequence of samples that asymptotically approximates the true posterior distribution. It allows for uncertainty quantification and inference based on the posterior samples. In practice, 10,000 samples were generated as burn-in, and the remaining 20,000 samples were thinned to keep every 20th sample to reduce serial correlation. Hence, 1,000 samples were collected to approximate the posterior.

We took the latest 334 new lines collected during the 2017/2018 season as the test population, and the remaining 1,051 lines collected prior to 2017 made up the training population. This is based on the comprehensive methodology we have described, trained, and tested previously with Australian cotton populations (Li Z. et al., 2022) in which the phenotype data have been processed and the training set was collected over several years and factors such as year and sampling batch have been corrected (Equation 1). The Pearson correlation between the genomic estimated and true phenotypes of the test population was defined as prediction accuracy. The prediction method was implemented and repeated 60 times, and the average prediction accuracy was calculated to reduce the randomness introduced by the MCMC sampling. On the replicates of these prediction accuracies, a *t*-test was conducted to judge whether the model performance is significantly different, with significant threshold set to be 0.05.

## Results

### DE results from key fibre RNA-seq analyses provided a large target gene list and a range of SNPs

Across the three key developmental stages of Coker315-C11 seed fibre, the pairwise DE analyses found that there were about 3,000 to 8,000 differentially expressed genes (at a stringent *p*-adjusted value <0.001) (**Supplementary File 1**). These findings are within the ballpark reported in detail regarding gene classes and pathways using a *G. raimondii* reference genome available at the time (MacMillan et al., 2017) and also found here but instead using the more recent *G. hirsutum* reference genome.

The identified gene classes span TFs, cell wall biosynthetic genes, multiple metabolic and structural genes, and unknown function genes; these have been examined in significant detail in terms of functional biological significance in terms of cotton fibre development by MacMillan et al. (2017). The fibre7DPA vs. fibre16DPA comparison (primary vs. transition wall stage comparison) identified 2,928 differentially expressed genes, with 1,487 upregulated and 1,441 downregulated genes, with log fold changes ranging from 13 to 2. These include NACs, MYBs, and arabinogalactan proteins including FLAs, auxin response genes, and many other unknown function genes. The fibre16DPA vs. fibre25DPA (transition vs. secondary wall stage comparison) comparison identified 3,917 differentially expressed genes, with 1,956 upregulated and 1,961 downregulated, with log fold changes ranging from 16 to 2. These include MYBs, NACs, phenylpropanoid pathway genes, FLAs, cellulose biosynthetic genes, auxin response genes, and many others including unknown function genes. The fibre7DPA vs. fibre25DPA (primary vs. secondary wall stage comparison) identified 7,678 differentially expressed genes, with 3,931 upregulated and 3,747 downregulated, with log fold changes ranging from 15 to 2. These include MYBs, NACs, lignin biosynthetic genes, FLAs, cellulose biosynthetic genes, auxin response genes, WRKYs, and many unknown function genes.

The Coker fibre DE gene lists enabled the identification of a suite of SNPs across the 63K SNP chip array of Hulse-Kemp et al. (2015), and these SNPs ranged in number across DE gene regions (**Supplementary File 2**). For the DE genes from the Fibre7DPA vs. Fibre16DPA comparison (primary vs. transition wall stage comparison), 85 SNPs were found within the gene-coding regions, 54 SNPs in the 1-kb gene-coding flanking regions, and 441 SNPs in the 10-kb gene-coding flanking regions. For the DE genes from the Fibre16DPA vs. Fibre25DPA comparison (transition vs. secondary wall stage comparison), 87 SNPs were found within the gene-coding regions, 72 SNPs in the 1-kb gene-coding flanking regions, and 594 SNPs in the 10-kb gene-coding flanking regions. For the DE genes from the Fibre7DPA vs. Fibre25DPA comparison (primary vs. secondary wall stage comparison), 260 SNPs were found within the gene-coding regions, 135 SNPs in the 1-kb gene-coding flanking regions, and 1,183 SNPs in the 10-kb gene-coding flanking regions. It should be noted that while the cotton genome contains ~80K protein coding genes, and a small subset of ~3K to 7.7K genes were found to be DE in the fibre development series used here, a small number of SNPs from the 63K array, in the order of hundreds (not several thousands or millions), were identified for GP accuracy tests.

The SNPs identified in DE fibre genes (of the variety of Coker 315-C11) were then tested for enhancing GP accuracy of fibre traits in a large multi-year multi-population GP model. In these tests, the SNPs were tested in three different scenarios where SNPs were preferentially weighted, as follows.

#### Weighted SNPs from DE genes alone did not enhance GP accuracy for fibre quality traits (Scenario 1)

**Figure 2:**
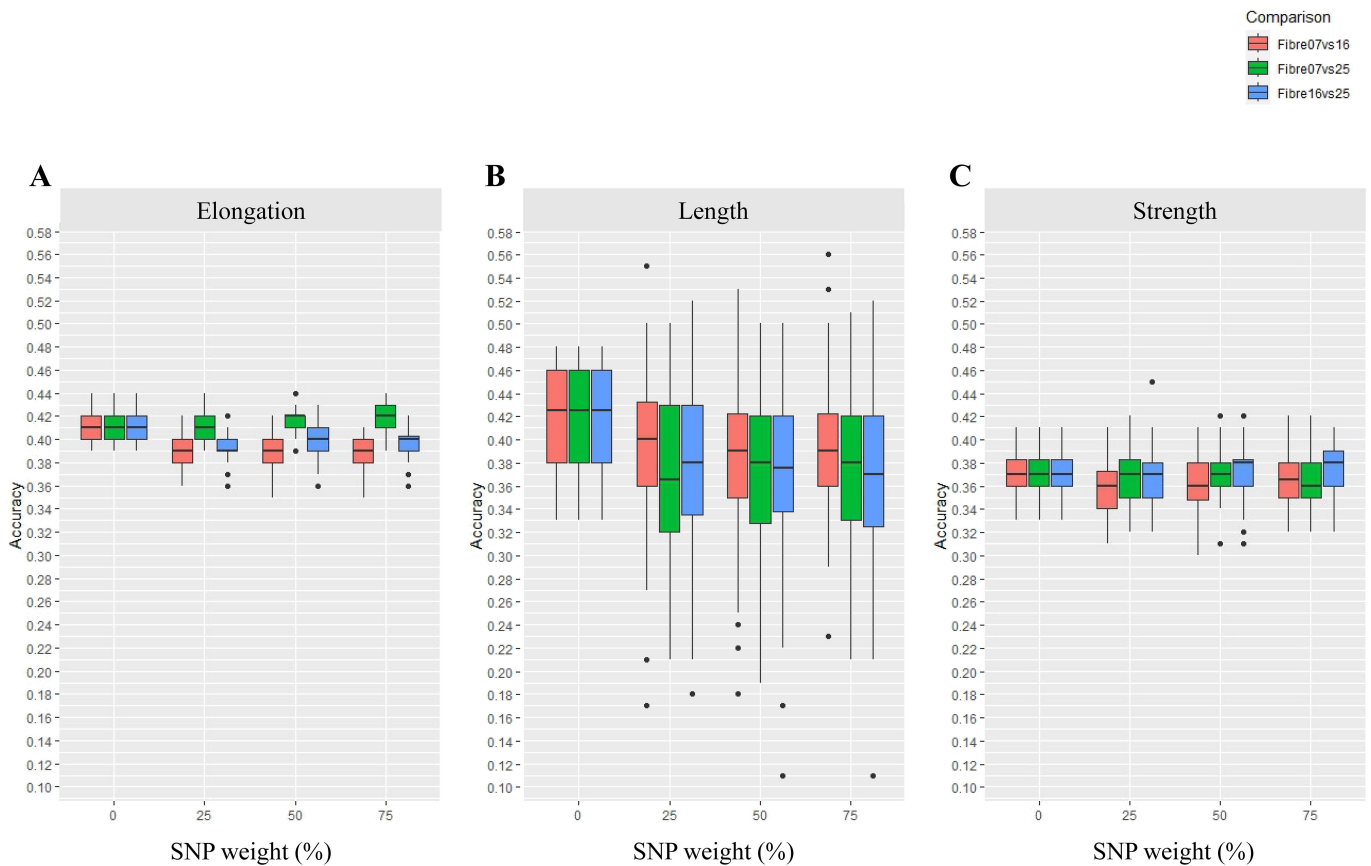
Accuracy of genomic predictions for fibre quality traits of **(A)** elongation, **(B)** length, and **(C)** strength using a DE gene approach with variable SNP weighting (Scenario 1). In this scenario, SNPs were weighted based on their coordinates in DE genes across three different fibre developmental stages (fibre 07 vs. 16, fibre 07 vs. 25 and fibre 16 vs. 25 DPA). The SNPs were weighted at 0%, 25%, 50%, and 75%, where 0 is unweighted SNPs in the GP model, or additional levels of weight is given in the GP model to target SNPs. The boxes show GP accuracy for fibre 07 vs. 16, fibre 07 vs. 25, and fibre 16 vs. 25 DPA in red, green, and blue, respectively. Each box shows 50% of the data range. A horizontal bar within each box shows the median. Vertical bars extending from each box shows the range of the remaining data. Dots indicate outliers.

The first scenario tested was weighting of SNPs in DE genes alone (**Figure 1B**), and with range of SNP weightings, from 0%, 25%, 50%, and 75%, tested. The result of weighting SNPs from the DE genes alone across three fibre development stages (fibre 07 vs. 16, fibre 07 vs. 25, and fibre 16 vs. 25) for estimating GP accuracy is presented in **Figure 2**; the summary results of the prediction accuracies are presented in **Supplementary File 3**. The SNPs used in this scenario are the complement of SNPs described above, which were identified across the DE genes.

Using SNPs in the DE genes alone was largely ineffective for enhancing the GP accuracy of cotton fibre traits tested. For example, the accuracy of GP showed little or no improvement for elongation (**Figure 2A**), and this was the case whether the SNPs in the DE genes were weighted at 25%, 50%, or 75%. In fact, for elongation, a reduction in GP accuracy was found in this DE-alone test scenario, for example, with the fibre 07 vs. 16 DE genes and to some extent also the fibre 16 vs. 25 DE genes, and this was evident for all SNP weightings. The lack of improvement of GP accuracy using DE genes alone also extended to the trait of length (**Figure 2B**) across all the SNP weighting tested. Furthermore, reduction in GP accuracy with DE genes alone was noticeable for length across all three developmental comparisons conducted, i.e., fibre 07 vs. 16, fibre 07 vs. 25, and fibre16 vs. 25, and as compared to the scenario where no weight was considered; in this case, the results were largely distributed, representing outliers, which might infer that the results were not reliable. For the trait of strength, using DE genes alone also did not enhance GP even when the SNPs in DE genes were weighted up to 75% (**Figure 2C**) and irrespective of the time point comparisons tested. On the other hand, in one instance, weighting of SNPs enhanced GP accuracy above the base level, and this was seen for elongation only with the fibre 07 vs. 25 DE genes and only at 75% SNP weighting; this improvement did not extend to the GP accuracy of length or strength and across any of the developmental comparisons tested.

### Weighted SNPs based on the annotation of targeted DE genes affected GP accuracy only when large gene regions were included (Scenario 2)

**Figure 3:**
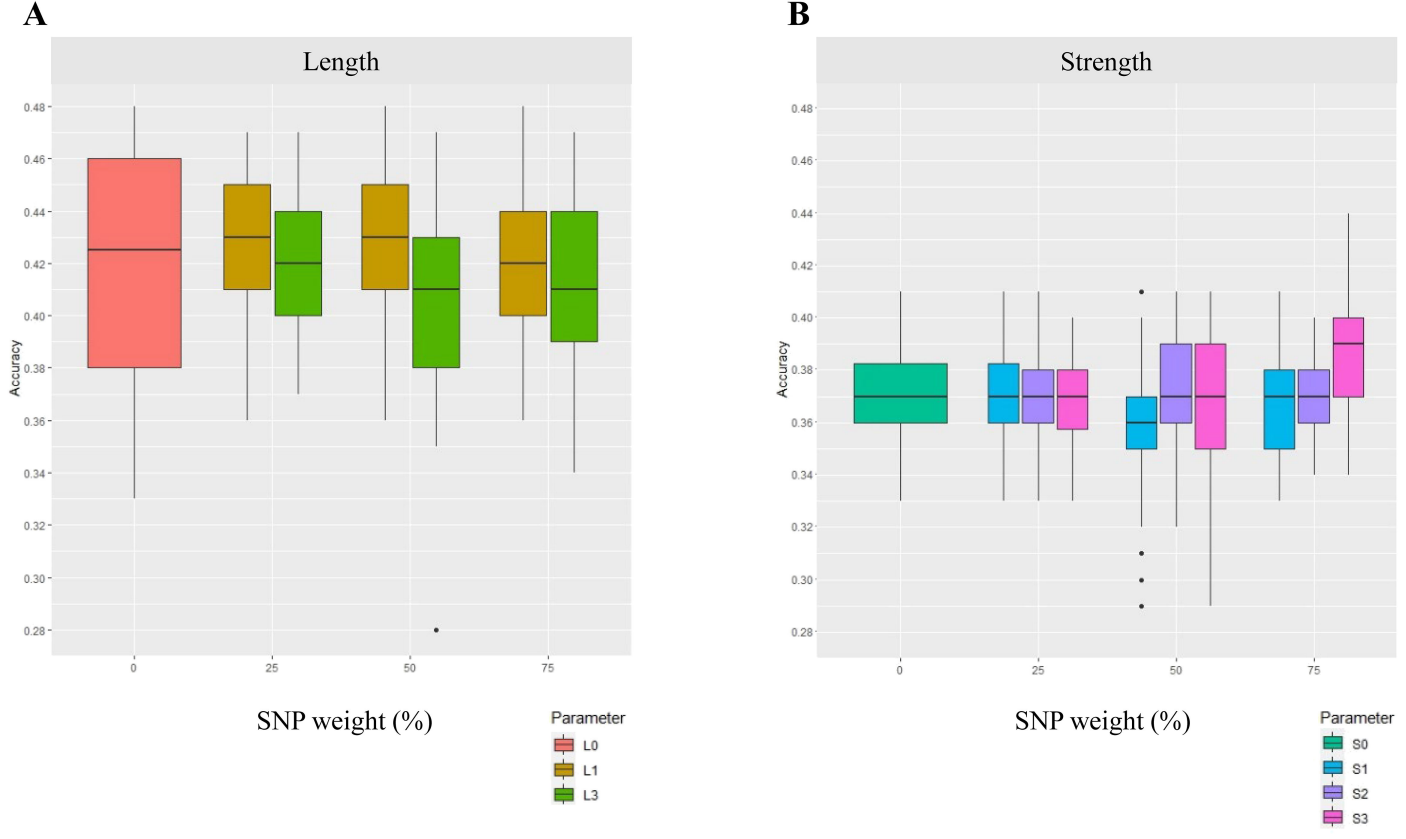
GP accuracy for fibre quality traits of **(A)** length and **(B)** strength, using a select DE gene approach and with SNP weighting (Scenario 2). In this scenario, SNPs were weighted based on their coordinates in select DE genes. The DE genes were from fibre developmental points of fibre 07 vs. 16 DPA for strength and fibre 07 vs. 25 DPA for length. The results are presented for SNP weights of 0%, 25%, 50%, and 75%. The legends show L and S for length and strength respectively. The numbers 0, 1, 2, and 3 indicate (0) unweighted SNPs, and where SNPs were available in (1) select DE gene-coding regions; (2) select gene-coding regions and within 1-kb flanking regions; and (3) select gene-coding regions and within 10-kb flanking regions, where the select DE genes were associated with down- and upregulated data sets of fibre developmental stages. No unique SNPs were found in the region L2, and hence no results were reported.

The second scenario tested in our workflow was the accuracy of GP using DE genes (**Figure 1B**) in conjunction with their annotation. The target traits tested were length and strength. The DE genes were targeted based on known function in relation to these traits. These include downregulated genes at fibre 07 vs. 16 and upregulated fibre 07 vs. 25 because these time points contribute to when cotton fibre cells develop some of their quality attributes. Within this scenario, we also tested whether the proximity of the SNPs to the target DE genes could increase GP accuracy, including within the gene-coding regions (i.e., the “exact” location), up to 1 kb flanking and including the gene regions, and up to 10 kb flanking and within the gene-coding regions. The results are shown in **Figure 3**. There was a small number of SNPs identified in the DE gene regions, with three to six SNPs in the 1-kb gene-coding regions, and up to 33 SNPs identified in the largest regions tested, i.e., the 10-kb regions of the DE genes. A summary statistic of accuracies and number of weighted SNPs in each sub-scenario is provided in **Supplementary File 4**.

The use of DE gene annotation for weighting SNPs changed GP accuracy only when a large enough region was included and SNPs were highly weighted (**Figure 3**). For strength, for instance, a 5.4% increase in GP accuracy was found for strength when SNPs in gene regions up to 10 kb of upregulated DE genes from fibre 07 vs. 25 were used (**Figure 3B**; **Supplementary Figure S3**, 75% weighting); SNPs in the exact or up to 1 kb flanking the gene region had a neutral effect (no effect) on GP accuracy and weighting of 25% or 50% also had no effect. For length, a very marginal GP enhancement was seen only when SNPs within and up to 1 kb of the target DE genes were used and only with 25% and 50% weighting. In fact, a downward shift of GP accuracy for length using the select DE gene approach was found if a larger region of SNPs up to 10 kb was used and particularly if weighted at 75% (**Figure 3A**).

### PCIT-based GCN clusters for SNP weighting improved GP accuracy across traits, particularly with FLA-based clusters of up to 3rd network neighbours (Scenario 3)

The third scenario tested in our workflow was highly targeted gene lists for trait-SNP identification generated using PCIT-based GCN clusters, and these included a nested gene network neighbour approach centred around key genes informed by biological function (**Figure 1C**). The PCIT-based GCN in this scenario was filtered to generate GCN clusters centred around the target FLA genes (FLA7, FLA11, and FLA12), and across their 1st, 2nd, and 3rd network neighbours for each GCN cluster.

On average, for each FLA GCN cluster generated, approximately 300 (1st), 1,200 (2nd), and 1,900 (3rd) neighbours were identified in each GCN gene cluster (**Supplementary File 5**). In each case, these specific GCN clusters included an intriguing range of TFs, cell wall biosynthetic genes, other genes of known and unknown function, as well as other FLAs (**Supplementary File 5**, individual lists); the FLA7, FLA11, and FLA12 GCN clusters with up to the 3rd network neighbours of each cluster were not identical and contained unique sets of genes as well as ~2,000 genes in common, and these included TFs and biosynthetic, cytoskeletal, and unknown function genes. The number of SNPs identified in the target genes identified here in scenario 3 was small—in the order of ~30 SNPs.

**Figure 4:**
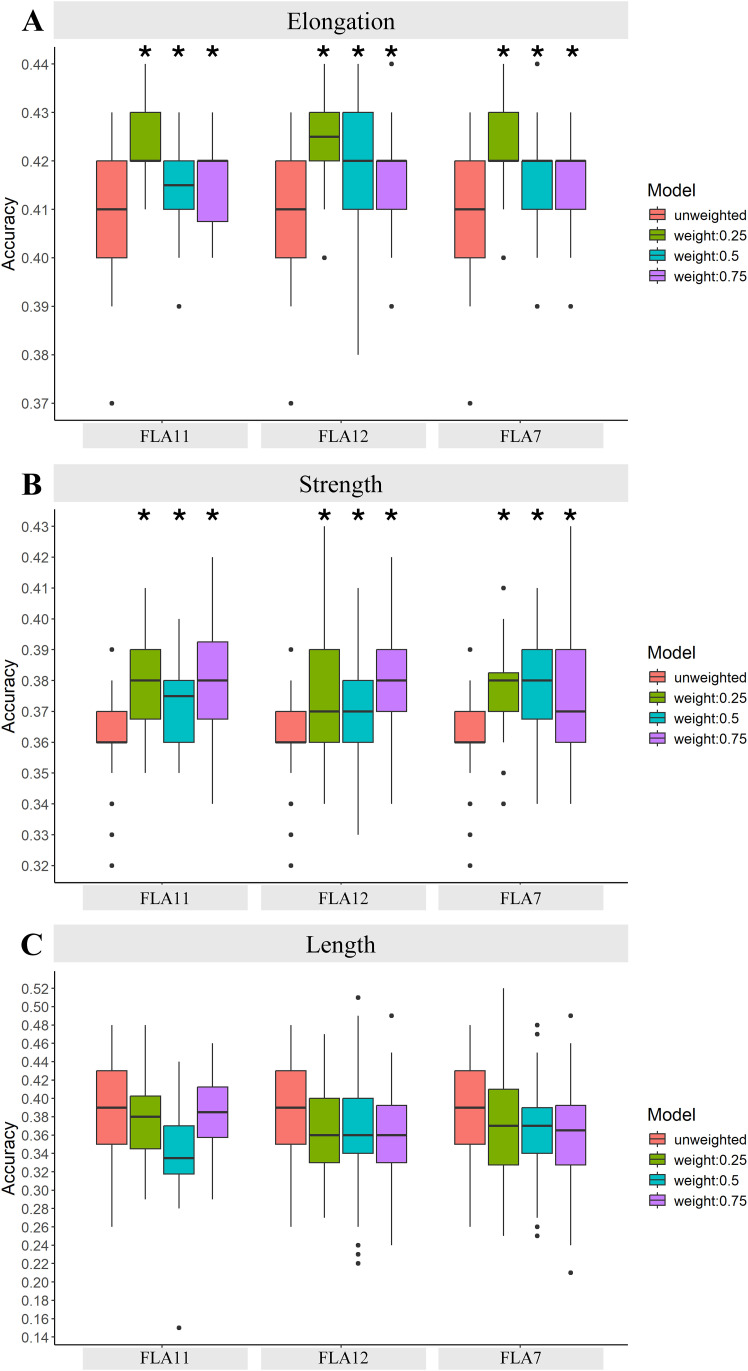
GP accuracy for fibre quality traits of **(A)** elongation, **(B)** strength, and **(C)** length using PCIT-based GCN clusters (Scenario 3). In this scenario, PCIT GCN clusters were generated centred on the key plant-fibre biomechanics genes of FLA7, FLA11, and FLA12. Here, for each FLA cluster, the first, second, and third neighbours of each FLA-based GCN cluster was tested for GP accuracy across the fibre traits of elongation, strength, and length. SNPs were weighted based on their coordinates in the selected DE genes in the network clusters from the fibre 16 vs. 25 DPA comparison. The results were presented for SNP weighted at 0%, 25%, 50%, and 75%. Asterisks indicate statistically significant differences at *p-*values >0.05 compared to the unweighted GP accuracies, i.e., with the standard Bayes C model; **Table 1** provides further details of the exact *p-*values.

Improved accuracy of GP was found by using GCN clusters and weighted SNPs, particularly when including up to the 3^rd^ network neighbours in each cluster (**Figure 4**; **Table 1**; **Supplementary File 6**). These results were evident across all three sets of GCN clusters tested, i.e., clusters centred around FLA7, FLA11, or FLA12. The increases in GP accuracy with each of the FLA GCN clusters was evident for elongation and strength as compared to unweighted scenarios. The use of FLA11 GCN clusters that included 1st, 2nd, and 3rd neighbours improved GP by 4.6% over that for unweighted GP for fibre elongation (**Figure 4A**), whereas inclusion of only the 1st neighbour had no significant effect (**Table 1**; **Supplementary File 6**). A similar result was also seen with the FLA12 GCN cluster with 4.5% improvement, and FLA7 GCN cluster with 3.4% improvement, for elongation over unweighted SNPs (**Figure 4A**). Increased GP accuracy using the FLA GCN clusters was also found for strength, and in this instance, up to 4.7%, when up to the 3rd network neighbours were included. The weighted SNPs in the genes from the FLA11 GCN cluster gave a 4.7% increase in strength GP accuracy (**Figure 4B**). Similarly, the FLA12 GCN cluster gave a 4.6% increase in strength GP accuracy with weighted SNPs (**Figure 4B**). The FLA7 GCN clusters with up to the 3rd network neighbours gave a 3.6% increase in GP accuracy for strength (**Figure 4B**). In general, weighting of the highly targeted SNPs at 50%, or 75% did not provide additional GP accuracy for elongation over that seen with the up to 3rd network neighbour approach (**Figure 4**; **Table 1**; **Supplementary File 6**).

**Table 1:** The *p-*values of the *t*-tests to compare the prediction performance between the Standard Bayes C model, and the Bayes C model incorporating the specific priors (weights) for a subset of SNPs relevant to the GCN clusters centred on the key plant-fibre biomechanics genes of FLA7, FLA11, and FLA12 for traits including elongation and strength in Scenario 3.

Gene	Neighbour	Trait	Weight = 0.25 vs. baseline	Weight = 0.5 vs. baseline	Weight = 0.75 vs. baseline
FLA7	1st N	EL	0.78	0.79	0.78
		STR	**2×10^-16^**	**7×10^-16^**	**2×10^-16^**
	2nd N	EL	**1×10^-10^**	**4×10^-9^**	**2×10^-8^**
		STR	0.37	0.22	**4×10^-4^**
	3rd N	EL	**6×10^-13^**	**1×10^-7^**	**8×10^-6^**
		STR	**2×10^-3^**	**4×10^-4^**	**6×10^-3^**
FLA11	1st N	EL	0.54	0.07	0.40
		STR	**5×10^-8^**	**1×10^-8^**	**1×10^-10^**
	2nd N	EL	**4×10^-10^**	**2×10^-7^**	**5×10^-9^**
		STR	**0.01**	**0.03**	**2×10^-8^**
	3rd N	EL	**1×10^-14^**	**2×10^-3^**	**3×10^-3^**
		STR	**6×10^-5^**	**5×10^-3^**	**7×10^-5^**
FLA12	1st N	EL	**0.01**	0.33	**0.03**
		STR	0.18	0.40	0.76
	2nd N	EL	**2×10^-11^**	**9×10^-8^**	**4×10^-7^**
		STR	**1×10^-5^**	**6×10^-8^**	**7×10^-9^**
	3rd N	EL	**3×10^-14^**	**6×10^-8^**	**2×10^-7^**
		STR	**1×10^-4^**	**0.02**	**8×10^-9^**

Bold values indicate statistically significant differences.

No change in GP enhancement was found for the trait of length (**Figure 4C**). In addition, the use of the FLA7, FLA11, and FLA12 PCIT-based GCN clusters also did not improve GP micronaire, short fibre index, and uniformity (**Supplementary File 6**). This indicates how critical biologically relevant genes and time points are for GP accuracy enhancement. The FLA7, FLA11, and FLA12 genes on their own are not known to be involved in controlling length, micronaire, short fibre index, or uniformity, i.e., are not specific to these traits and so this result was somewhat expected and also shows the importance of our approach in using important genes and gene network clusters associated with a particular trait. Furthermore, the developmental time points used in this scenario (fibre 16 vs. 25 DPA) are beyond when most of the fibre’s growth to its final length (~fibre 12 to 14 DPA depending on the species) has occurred and thus no key genes enriched for controlling the trait of length would be present in the DE/PCIT analyses.

The computational efficiency of this method was the same as in the original Bayes C model, since the transcriptome data were merely used as a prior (Li Z. et al., 2022).

### Improvements in GP accuracy across the three scenarios

Here, three scenarios were tested for improving GP accuracy. The PCIT-based GCN approach (Scenario 3) provided the best improvements for GP accuracy, compared to the other approaches tested (Scenarios 1 and 2). For example, the PCIT-based GCN approach to weight SNPs improved GP accuracy of fibre elongation by 4.6%, and strength by 4.7% (**Figure 4**). This is in contrast to weighting SNPs using all the DE genes (Scenario 1; **Figure 2**), which largely did not improve GP for elongation, strength, or length. In addition, when targeted DE gene lists were used (Scenario 2; **Figure 3**), there was only GP accuracy improvement with the use of a large gene region of 10 kb and only in the case of fibre strength with a GP accuracy increase of 5.4%; no increases were seen for elongation or length, or smaller gene regions for strength.

The Bayesian model that incorporated the information of GCN clusters produced significantly better prediction accuracies compared to the standard Bayes C model (**Table 1**, probability statistics based on *t*-tests), i.e., by giving more weights in the priors to a subset of SNPs that are associated with neighbours of three fibre development-related genes.

The PCIT-based GCN approach was more effective than the other methods in improving GP accuracy. Like Scenarios 1 and 2, it also generated large lists of DE genes—in the order of hundreds to thousands (**Supplementary Files 1**, **2**, **5**). However, the PCIT-based GCN approach used genes with known biological function related to the trait of interest as the entry point basis of identifying other co-expressed genes (**Supplementary File 7**). These co-expressed genes include genes that either are in known pathways, have no known function, have not been substantively functionally tested, have functions potentially linked to the traits of interest, or have functions beyond what is currently known for the traits of interest. With this PCIT-based GCN cluster approach, a targeted set of genes for the traits of interest was identified in a novel way from the large transcriptome data set, and the very large genome. The other scenarios did not take such a biology-informed and targeted approach.

### GCN clusters for improved trait GP have known and unknown fibre strength and elongation genes

The three GCN clusters (with 1st, 2nd, and 3rd neighbours) that improved GP accuracy for fibre strength and elongation had in common a suite of TFs, glycoproteins, transporters and membrane anchored/trafficking, defence signalling, enzymes, and unknown function (**Supplementary File 7**). Several MYBs included those with a known role in the plant SCW pathway such as MYB61, MYB43, MYB54, and other MYBs (Xiao et al., 2021). Homeodomain TFs included KNAT7 homeologues and several BEL-1-like 2 and BEL-1-like 6, plus additional homeobox proteins. MAD TFs include AGL8-like and AGL30-like. NACs included NAC58. Other TFs included tri-helix GT-2s and zinc-finger binding proteins. Glycoproteins included SCW FLAs, which are known to have roles in plant fibre biomechanics, lectins such as an F-box phloem protein 2, other hydroxy-proline rich glycoproteins, an extensin, and a LEA-2. In addition to transporters, several membrane/vesicle trafficking proteins included clathrin-related, synaptotagmin, and a tetraspannin-14-like. Several lipid transfer proteins (LTP-2) also featured. A leucine-rich-repeat protein and a pathogenesis-related protein 5 were amongst the defence/stress-pathway genes. Enzymes involved in polysaccharide synthesis and cell wall polymer formation included a sucrose synthase, various pectinesterases/pectinesterase inhibitor-likes, cytochrome P450 monoxygenase ferulate-5-hydroxylases (F5H), a flavanone 3-dioxygenase, as well as chitinases and, interestingly, also a cucumisin, a serine peptidase found in plants.

## Discussion

This research explored the potential integration of plant-biology-based transcriptomic information derived from RNA-Seq analysis into GP models for cotton breeding (**Figure 1**). The study focused on elucidating key genes and gene networks associated with valuable fibre traits—such as length, elongation, and strength—to inform more accurate GP. Leveraging DE analysis across three crucial developmental stages of seed fibre, numerous genes were identified, revealing thousands of DE genes across these stages. These genes span various classes, including TFs, cell wall biosynthetic genes, and structural genes, shedding light on their crucial roles in fibre development. Integration of this transcriptomic data with genomic information allowed for the identification of SNPs located within or near specific DE genes. However, attempts to directly add more weight on these SNPs in the GP model based on DE genes or their annotations did not notably improve GP accuracy. In contrast, when incorporating information from PCIT-based gene networks derived from DE genes and their 1st, 2nd, and 3rd neighbouring nodes, the GP model exhibited enhanced accuracy for predicting elongation and strength. In other words, use of the latter scenario (Scenario 3) led to ~5% increases, whereas Scenarios 1 and 2 were not as effective. The accuracy for predicting fibre length showed a reduction, possibly owing to the nuances of the chosen developmental stage comparison and the genes used to generate the network clusters. This comprehensive approach offers insights into potential candidate genes and networks that impact cotton fibre traits, laying a groundwork framework and a feasible approach for more refined and accurate GS strategies in cotton breeding.

PCIT-based GCN clusters were effective in improving GP accuracy for cotton fibre quality traits. This was evident with a 4.6% improvement for elongation and a 4.7% for strength GP accuracy for cotton fibre quality. Inclusion of both the PCIT and the target GCN approaches together proved valuable and opens up opportunities for advancing precision breeding in cotton using such an approach. PCIT as an approach has also been of value in studies of animals and humans, with key regulator genes being identified as predictors controlling ~500 genes associated with heifer puberty (Nguyen et al., 2018), significant suites of bovine fertility genes identified such as regulatory and functional genes as well as non-coding RNAs (Fonseca et al., 2022), and pig gut microbiotal changes across intestinal tracts and their association with energy homeostasis (Crespo-Piazuelo et al., 2018), and in identifying key target genes in studies of human cancers (Li D. et al 2022), asthma (Banerjee et al., 2021), and the SARS-CoV-2/human interactome (Guzzi et al., 2020). In this present study of cotton and the PCIT-based GCN approach, a small number of SNPs led to GP improvement: ~30 SNPs. Together, this points to future opportunities that can be tested for identifying and testing additional GCN clusters and hence additional valuable gene clusters and beneficial SNPs for enhancing GP accuracy in crops. For future work, additional key genes can be explored given the promising findings here with FLAs. Furthermore, stringent criteria were used in our DE analysis; in the future, reducing stringency may then include additional genes in the networks, and this could be tested to see if more prediction accuracy can be achieved. In addition, large gene regions in GP accuracy is another area for further exploration. For example, linkage disequilibrium (LD) is a challenge for GP and GS, and several studies have explored this challenge especially where multiple SNPs can exist in a large LD block, for example, in cotton (Li et al., 2024). Here, in our current study, the finer resolution within large gene regions has been achieved for identifying SNPs through biology-informed gene networks and PCIT.

The targeted GCN scenario (PCIT-based) improved GP accuracy best with up to 3rd neighbours and flags potential utility in cotton breeding. Such a gene network neighbour clustering approach for GP in crop breeding is, to our knowledge, not currently being used, but our findings provide practical evidence and workflows to help achieve the vision and aspiration for future breeding such as large gene-to-phenotype networks that others have pointed out (e.g., Powell et al., 2022). With only three traits tested here across the various scenarios, our findings open up the possibility for further traits that could be more accurately predicted for cotton fibre and other traits. Future improvements for GP accuracy of cotton could include incorporating additional transcriptomic data from biologically relevant time points, for example, earlier development time points that enrich for genes controlling the trait of length. Given the advancement described here with our approach, future studies could investigate whether giving specific weighting to non-synonymous SNPs and those within promoter regions could enhance prediction accuracy; this is possible with the Bayesian model. In addition, our approach also points to how large-scale target SNP populations can be identified for precision breeding.

The suite of FLA GCN clusters linked to fibre strength and elongation provides further insights into the molecular biology of the construction unique cotton fibre cell wall for which several genes and pathways have identified (MacMillan et al., 2017; Wang et al., 2017; Ma et al., 2018; Li et al., 2020; Gallagher et al., 2020; Li et al., 2021a; Yang et al., 2023); this also extends potentially to fibre colour. The SCW pathway TFs such as KNAT-7/BEL-1like homeodomain TFs in the GCN clusters in this research are known to modulate cotton fibre cell growth in different ways, and their expression level may have an impact on increased or decreased strength and elongation traits (Jia et al., 2024; Li et al., 2011). In addition to the known plant glycoproteins affecting strength and elongation such as FLA12 and FLA11 (MacMillan et al., 2010, 2015, 2017; Ma et al., 2022), it is interesting to note that other genes appear to also contribute. For example, F-box phloem protein 2 type proteins, which are starting to be functionally characterised in cotton (Wei et al., 2023), may point to potential recruitment of these genes to the cotton seed fibre development in addition to roles in phloem. Similarly, the extensin Pollen_Ole_e_like in the GCN clusters has been implicated with selenium binding proteins in Arabidopsis and potentially involved in ROS signalling (Dervisi et al., 2023) and may contribute to how the cell wall properties of strength and elongation are formed in the cotton fibre cell. When the fibre generates its thick highly cellulosic cell wall, multiple events are in play such as membrane/vesicle trafficking and signalling. During this process, the glucose required for cellulose synthesis, extracellular matrix remodelling, and a high energy load requires sucrose synthases and pectinesterases/pectinesterase inhibitor-likes that were found in the GCNs here. As cotton seed fibres are essentially composed of highly cellulosic SCWs without lignin (MacMillan et al., 2013, 2017), the results here point to potential redirection of the flavonol pathway in cotton fibres away from lignin-specific monomers potentially via cytochrome P450 monoxygenase ferulate-5-hydroxylases and a flavanone 3-dioxygenase, and potentially also via regulation through NAC58, which affects suberin deposition in cotton fibres (Nomberg et al., 2022). Valuable SNPs in these genes in particular populations are therefore potentially useful for GP for breeding.

Using a DE gene-only approach was not effective in enhancing GP accuracy in this study even with varying the extent of SNP weighting; this may point to the complexity of genome to phenome in crop development and target traits such as in cotton. Based on this, only using DE genes or their annotation information to weight SNPs may not be sufficient to enhance GP accuracy, and so future studies for enhancing GP accuracy only using DE genes may require other methods or data integration approaches to optimise models. In terms of DE gene studies for cotton fibre improvement, over the last 5 years, dozens of articles have been published internationally, and generally, these report substantial sets of DE genes with potential for impacting fibre quality. For example, these include studies combining GWAS and eQTLs to identify fibre cell wall development regulatory networks (Li et al., 2020), co-expression analyses for genes differentially expressed with high cotton fibre quality (Zou et al., 2019), and high-density genetic map studies for QTLs and genes for fibre quality and yield in cotton analysis of vegetative to reproductive transition and branching genes linked to planting density (Gu et al., 2020) and also linked to natural colour formation in fibres (Tang et al., 2021). In our study, weighting of SNPs increased their efficacy for GP accuracy depending on their location, and this could be due to the fact that negative and positive SNPs can co-occur across chromosomal regions, e.g., SNPs in enhancers, suppressor regions, and promoters; when such SNPs in key genes are important for a trait, then the interplay of positive and negative SNPs could come into effect. Given the results of this study, such DE lists likely hold a rich wealth of important insights into the genes underlying the biology of fibre formation and use in GP accuracy for traits of interest. In addition, using only trait-specific and not non-trait-specific genes is important for improving GP accuracy, and thus, the PCIT-based gene network cluster approach helps identify trait-specific genes that may not always be known.

The GCN approach on the basis of PCIT or other network inference approaches holds potential application for accelerating genetic gain in crops, through the improved GP accuracy, as results found here for cotton fibre quality traits. This could help with accelerating genetic gain of complex traits, which remains a challenge for crops, for example, in cotton (Conaty et al., 2022), and for which transdisciplinary approaches with other systems like livestock GS are helping expand this field (Voss-Fels et al., 2019). As an extension, integration of the GCN approach with GP and GS for improvements in a range of traits may hold potential for various crop traits such as yield improvement including grain yield, plant height, flowering time, and inflorescence traits, in crops such as wheat, maize canola, rice, and others. This could integrate with existing transcriptomics data sets and provide a complementary line of research with transcriptomics-wide associated studies and associated data sets, especially if there are functionally confirmed genes for a target trait that could be used as entry points into GCNs to generate targeted GCN clusters for testing of GP accuracy for desired traits. Such research requires broader testing of this GCN approach in additional traits and a broader range of germplasms. It could also explore testing the potential integration with genome by environment interactions for improving GP and GS. The positive findings here were based on multi-line phenotypic trait data across many years of field data and a transcriptomics series of an unrelated line grown in a glasshouse experiment with representative growth conditions. Future research could examine whether confounding factors may affect improved GP accuracy using the GCN approach, such as environmental factors and/or genetic background and population structure; these would require large data sets and experiments with different environments and genetic backgrounds. More advanced statistical models might also be needed to account for those confounding factors in both GCN and GP analyses.

Our strategy can be easily used in breeding practice. The transcriptome experiments and data analysis were done independently from the GP, and the outcome of transcriptome data analysis was used as prior information in Bayesian prediction models applicable for any cotton GP data set that targeted fibre qualities. This is a much simpler and cost-effective approach for a breeding program compared to current existing omics approaches that rely on measuring both the transcriptome and genomic data for thousands of samples (e.g., Hu et al., 2019; Azodi et al., 2020). The biology-informed GCN approach for GP of fibre quality traits can now be practically employed in GP and GS pipelines, as described in **Figure 1**. Valuable SNPs could be used in GS-based breeding pipelines for accelerating genetic gain of target traits and hence incorporation into new varieties. If the prediction accuracy is higher due to incorporating the gene network information, then the ranking of lines based on predicted phenotype scores will be more accurate, and then we have a better chance to find desired genotypes based on prediction results. As a future direction, evaluation will be required to determine whether this strategy can also be beneficial to the multiple environment GP analysis (i.e., prediction models with G×E interactions), which is essential from the application point of view in a plant breeding program.

In conclusion, this study found that integration of biology-informed RNA-seq data using PCIT-based GCNs for weighting SNPs improved GP accuracy for two important cotton quality traits, namely, fibre strength and elongation. The study also identified existing and potentially new genes involved in the formation of cotton fibre traits. This provides new insights and practical approaches for enhancing genomic assistance-based cotton breeding where precise identification of valuable SNPs that increase GP accuracy of an economic trait can be integrated in GS pipelines for cotton breeding. This study makes a unique contribution to cotton breeding by demonstrating the possibility of successfully integrating fibre developmental biology, transcriptomics, gene-networks, and GP models for accelerating the GS kind of knowledge, and future studies with additional time points and genes could accelerate the development of improved cotton varieties with enhanced fibre length, elongation, and strength, meeting the demands of the textile industry and ensuring the sustainability of cotton production. Potential areas for further investigation based on the findings reported here include functional studies of cotton fibre strength and elongation, SNP variants and how they affect trait formation, examination of how the additional “new/unknown” fibre genes may affect fibre development and traits, and whether/how environmental factors may be integrated in GCN for GP and GS of fibre traits. Breeding approaches such as those taken here for cotton remain to be explored in other crops.

## Data Availability

The cotton variety phenotype and genotype data are publicly available at the CSIRO Data Access Portal https://data.csiro.au/collection/csiro:54562. The cotton Coker 315-C11 seed fibre transcriptome data is available at the CSIRO Data Access Portal https://data.csiro.au/collection/csiro:63229.

## Supplementary material

Supplementary File 1 CokerFibreDevelopmentDEPairwiseLists.

Supplementary File 2 CokerFibreDEgenes_SNPcoordinates.

Supplementary File 3 Accuracy prediction estimates for three fibre life stage comparisons.

Supplementary File 4 Summary statistic of accuracy of genomic predictions for fibre quality traits.

Supplementary File 5 CokerPCITbasedGeneNetworks GP and Neighbours.

Supplementary File 6 GP of all 6 fibre quality traits with FLA7, FLA11, and FLA12 GCN-clusters, at the 1^st^, 2^nd^, and 3^rd^ network neighbour level in each cluster.

Supplementary File 7 Summary of genes common to FLA7, 11, 12 PCIT-based GCN clusters.
