# Supplementary material for: Leveraging transcriptomics-based approaches to enhance genomic prediction: integrating SNPs and gene networks for cotton fibre quality improvement
Source: Front Plant Sci. 2024 Sep 20;15:1420837. doi: 10.3389/fpls.2024.1420837 (PMC11450228; doi:10.3389/fpls.2024.1420837)
Supplement: Supplementary File 3 — Accuracy prediction estimates for three fibre life stage comparisons. [file Table3.docx]

**Supplementary table 3.** Accuracy prediction estimates for three fibre life stage comparisons (fibre 07 vs 16, fibre 07 vs 25 and fibre 16 vs 25) across three fibre quality traits (elongation, length and strength). The mean and standard deviation of accuracies are provided for different weighting schemes (0, 25, 50 and 75 %).

| **Comparison** | **Trait** | **Weight** | **Mean of Accuracy** | **Standard deviation of accuracy** | **Weighted SNPs** | **Total SNPs** | **Test**  **population** | **Total population** |
| --- | --- | --- | --- | --- | --- | --- | --- | --- |
| Fibre07vs16 | Elongation | 0 | 0.41 | 0.01 | 0 | 12296 | 334 | 1907 |
| Fibre07vs16 | Elongation | 25 | 0.39 | 0.01 | 85 |  |  |  |
| Fibre07vs16 | Elongation | 50 | 0.39 | 0.01 |  |  |  |  |
| Fibre07vs16 | Elongation | 75 | 0.39 | 0.01 |  |  |  |  |
| Fibre07vs25 | Elongation | 0 | 0.41 | 0.01 | 0 |  |  |  |
| Fibre07vs25 | Elongation | 25 | 0.41 | 0.01 | 165 |  |  |  |
| Fibre07vs25 | Elongation | 50 | 0.42 | 0.02 |  |  |  |  |
| Fibre07vs25 | Elongation | 75 | 0.42 | 0.01 |  |  |  |  |
| Fibre16vs25 | Elongation | 0 | 0.41 | 0.01 | 0 |  |  |  |
| Fibre16vs25 | Elongation | 25 | 0.39 | 0.01 | 87 |  |  |  |
| Fibre16vs25 | Elongation | 50 | 0.4 | 0.01 |  |  |  |  |
| Fibre16vs25 | Elongation | 75 | 0.4 | 0.01 |  |  |  |  |
| Fibre07vs16 | Length | 0 | 0.41 | 0.05 | 0 |  |  |  |
| Fibre07vs16 | Length | 25 | 0.39 | 0.05 | 85 |  |  |  |
| Fibre07vs16 | Length | 50 | 0.38 | 0.05 |  |  |  |  |
| Fibre07vs16 | Length | 75 | 0.39 | 0.07 |  |  |  |  |
| Fibre07vs25 | Length | 0 | 0.41 | 0.07 | 0 |  |  |  |
| Fibre07vs25 | Length | 25 | 0.37 | 0.07 | 165 |  |  |  |
| Fibre07vs25 | Length | 50 | 0.37 | 0.07 |  |  |  |  |
| Fibre07vs25 | Length | 75 | 0.37 | 0.07 |  |  |  |  |
| Fibre16vs25 | Length | 0 | 0.41 | 0.07 | 0 |  |  |  |
| Fibre16vs25 | Length | 25 | 0.37 | 0.06 | 87 |  |  |  |
| Fibre16vs25 | Length | 50 | 0.37 | 0.06 |  |  |  |  |
| Fibre16vs25 | Length | 75 | 0.37 | 0.07 |  |  |  |  |
| Fibre07vs16 | Strength | 0 | 0.37 | 0.02 | 0 |  |  |  |
| Fibre07vs16 | Strength | 25 | 0.36 | 0.02 | 85 |  |  |  |
| Fibre07vs16 | Strength | 50 | 0.36 | 0.02 |  |  |  |  |
| Fibre07vs16 | Strength | 75 | 0.36 | 0.02 |  |  |  |  |
| Fibre07vs25 | Strength | 0 | 0.37 | 0.02 | 0 |  |  |  |
| Fibre07vs25 | Strength | 25 | 0.37 | 0.02 | 165 |  |  |  |
| Fibre07vs25 | Strength | 50 | 0.37 | 0.02 |  |  |  |  |
| Fibre07vs25 | Strength | 75 | 0.37 | 0.02 |  |  |  |  |
| Fibre16vs25 | Strength | 0 | 0.37 | 0.02 | 0 |  |  |  |
| Fibre16vs25 | Strength | 25 | 0.37 | 0.02 | 87 |  |  |  |
| Fibre16vs25 | Strength | 50 | 0.37 | 0.02 |  |  |  |  |
| Fibre16vs25 | Strength | 75 | 0.38 | 0.02 |  |  |  |  |
