# Supplementary material for: Leveraging transcriptomics-based approaches to enhance genomic prediction: integrating SNPs and gene networks for cotton fibre quality improvement
Source: Front Plant Sci. 2024 Sep 20;15:1420837. doi: 10.3389/fpls.2024.1420837 (PMC11450228; doi:10.3389/fpls.2024.1420837)
Supplement: Supplementary File 4 — Summary statistic of accuracy of genomic predictions for fibre quality traits. [file Table4.docx]

**Supplementary 4.** Summary statistic of accuracy of genomic predictions for fibre quality traits (length and strength). It represents the mean and standard deviation of accuracies from down-regulated fibre 07 vs 16 for length and up-regulated fibre 07 vs 25 for strength using 0, 25, 50 and 75 % weights applied to SNPs associated with DE genes. L and S represent length and strength while 0, 1, 2 and 3 are indicatives of unweighted scheme, sub-scenarios with SNPs in exact, exact and within1 kb and, exact, within 1 kb and within 10 kb of DE genes.

| Trait | Weight | Parameter | Mean of accuracy | Standard deviation of accuracy | Weighted SNPs | Total SNPs | Test Population | Total population |
| --- | --- | --- | --- | --- | --- | --- | --- | --- |
| Length | 0 | L0 | 0.41 | 0.05 | 0 | 12296 | 334 | 1907 |
|  | 25 | L1 | 0.43 | 0.04 | 3 |  |  |  |
|  | 50 |  | 0.43 | 0.04 | 3 |  |  |  |
|  | 75 |  | 0.42 | 0.04 | 3 |  |  |  |
|  | 25 | L3 | 0.42 | 0.04 | 29 |  |  |  |
|  | 50 |  | 0.41 | 0.04 | 29 |  |  |  |
|  | 75 |  | 0.41 | 0.04 | 29 |  |  |  |
|  | 0 | S0 | 0.37 | 0.04 | 0 |  |  |  |
| Strength | 25 | S1 | 0.37 | 0.04 | 6 |  |  |  |
|  | 50 |  | 0.36 | 0.04 | 6 |  |  |  |
|  | 75 |  | 0.37 | 0.04 | 6 |  |  |  |
|  | 25 | S2 | 0.37 | 0.04 | 7 |  |  |  |
|  | 50 |  | 0.37 | 0.04 | 7 |  |  |  |
|  | 75 |  | 0.37 | 0.04 | 7 |  |  |  |
|  | 25 | S3 | 0.37 | 0.04 | 33 |  |  |  |
|  | 50 |  | 0.37 | 0.04 | 33 |  |  |  |
|  | 75 |  | 0.39 | 0.04 | 33 |  |  |  |
