# Supplementary material for: Leveraging transcriptomics-based approaches to enhance genomic prediction: integrating SNPs and gene networks for cotton fibre quality improvement
Source: Front Plant Sci. 2024 Sep 20;15:1420837. doi: 10.3389/fpls.2024.1420837 (PMC11450228; doi:10.3389/fpls.2024.1420837)

| Gene | Neighbour | Trait | Weight=0.25 VS Baseline | Weight=0.5  VS Baseline | Weight=0.75  VS Baseline |
| --- | --- | --- | --- | --- | --- |
| FLA7 | 1st N | EL | 0.78 | 0.79 | 0.78 |
|  |  | STR | **2×10^-16^** | **7×10^-16^** | **2×10^-16^** |
|  | 2nd N | EL | **1×10^-10^** | **4×10^-9^** | **2×10^-8^** |
|  |  | STR | 0.37 | 0.22 | **4×10^-4^** |
|  | 3rd N | EL | **6×10^-13^** | **1×10^-7^** | **8×10^-6^** |
|  |  | STR | **2×10^-3^** | **4×10^-4^** | **6×10^-3^** |
| FLA11 | 1st N | EL | 0.54 | 0.07 | 0.40 |
|  |  | STR | **5×10^-8^** | **1×10^-8^** | **1×10^-10^** |
|  | 2nd N | EL | **4×10^-10^** | **2×10^-7^** | **5×10^-9^** |
|  |  | STR | **0.01** | **0.03** | **2×10^-8^** |
|  | 3rd N | EL | **1×10^-14^** | **2×10^-3^** | **3×10^-3^** |
|  |  | STR | **6×10^-5^** | **5×10^-3^** | **7×10^-5^** |
| FLA12 | 1st N | EL | **0.01** | 0.33 | **0.03** |
|  |  | STR | 0.18 | 0.40 | 0.76 |
|  | 2nd N | EL | **2×10^-11^** | **9×10^-8^** | **4×10^-7^** |
|  |  | STR | **1×10^-5^** | **6×10^-8^** | **7×10^-9^** |
|  | 3rd N | EL | **3×10^-14^** | **6×10^-8^** | **2×10^-7^** |
|  |  | STR | **1×10^-4^** | **0.02** | **8×10^-9^** |

Supplementary File 6

1. FLA7 (1^st^ neighbour)


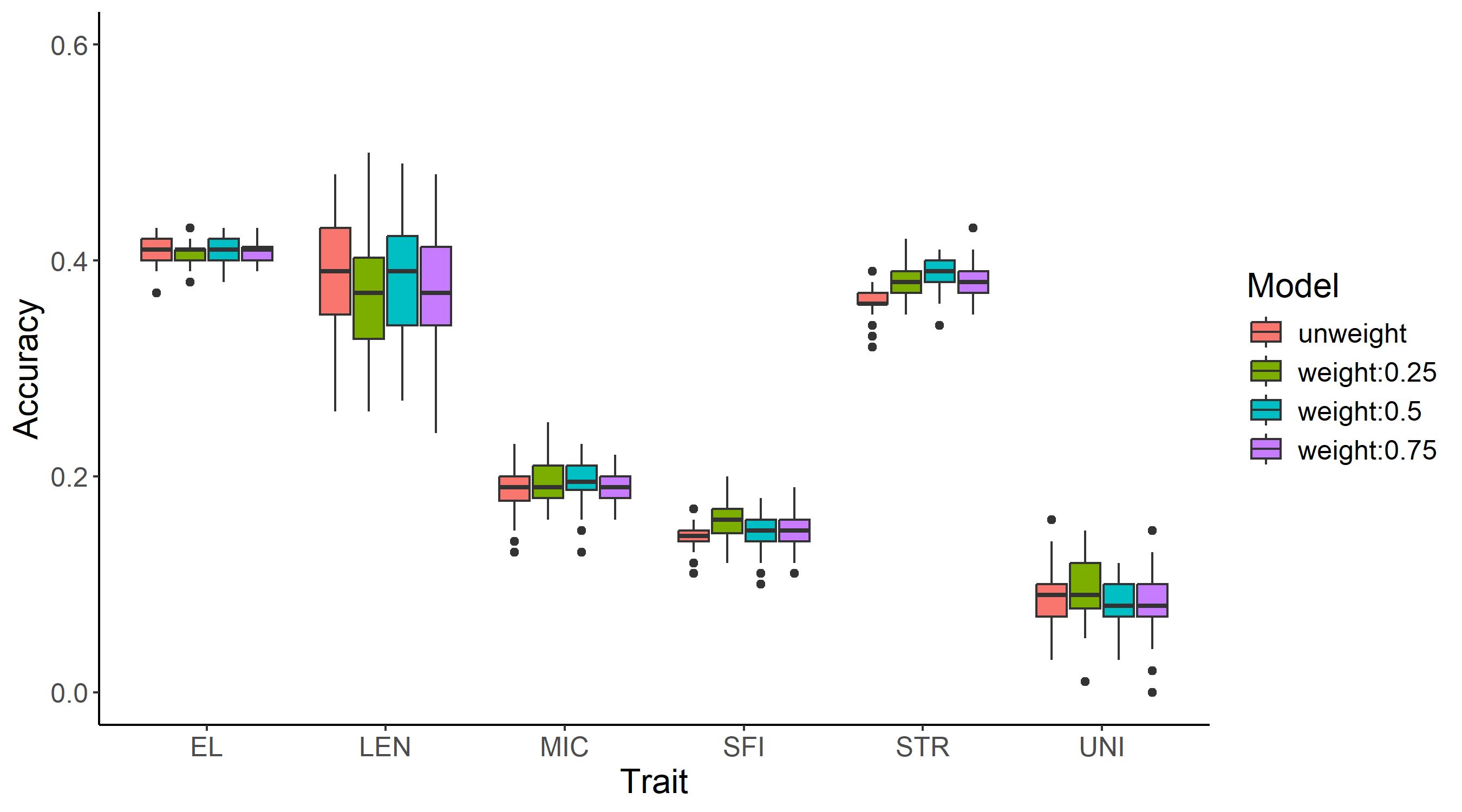


1. FLA7 (2^nd^ neighbour)


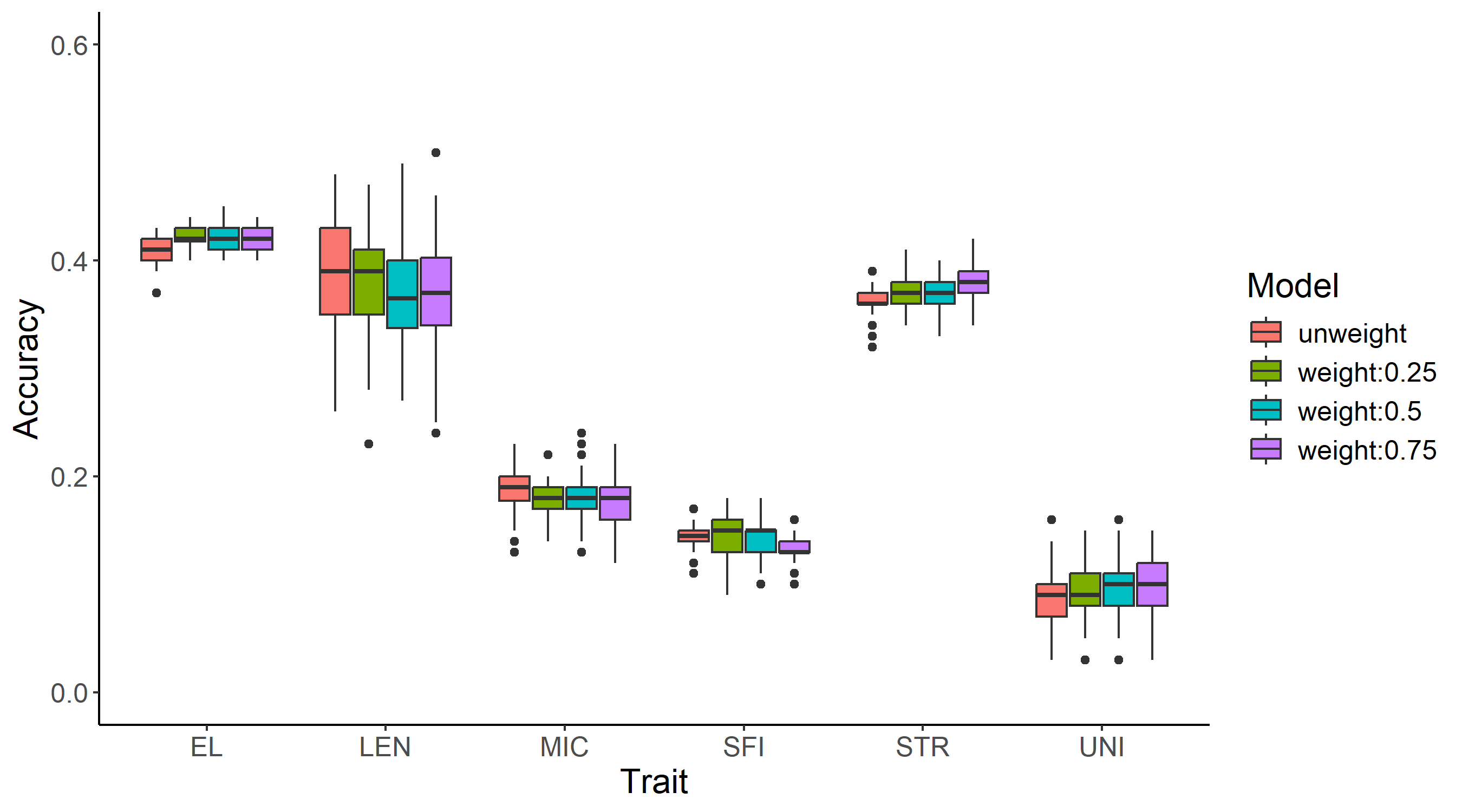


1. FLA7 (3^rd^ neighbour)


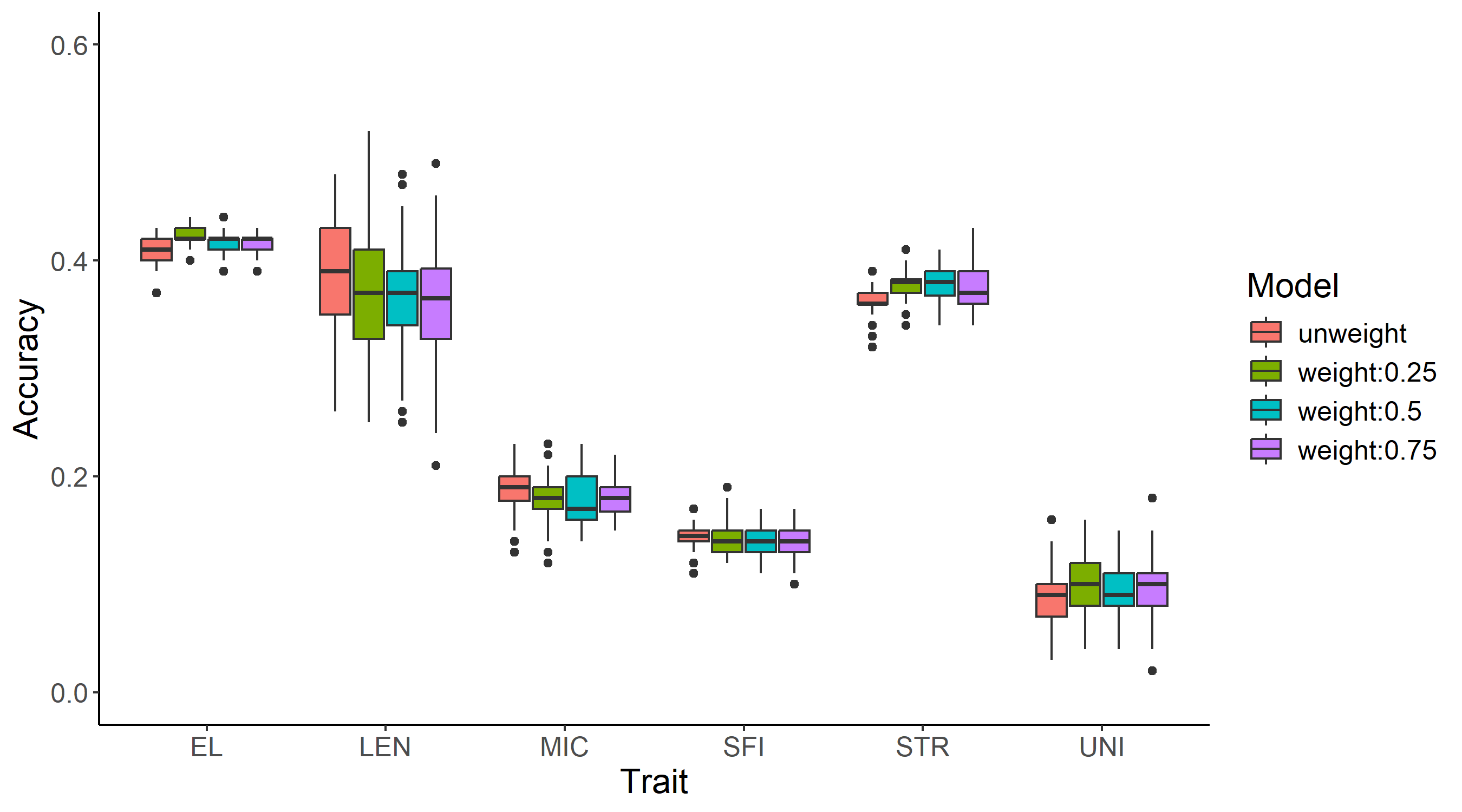


1. FLA11 (1^st^ neighbour)


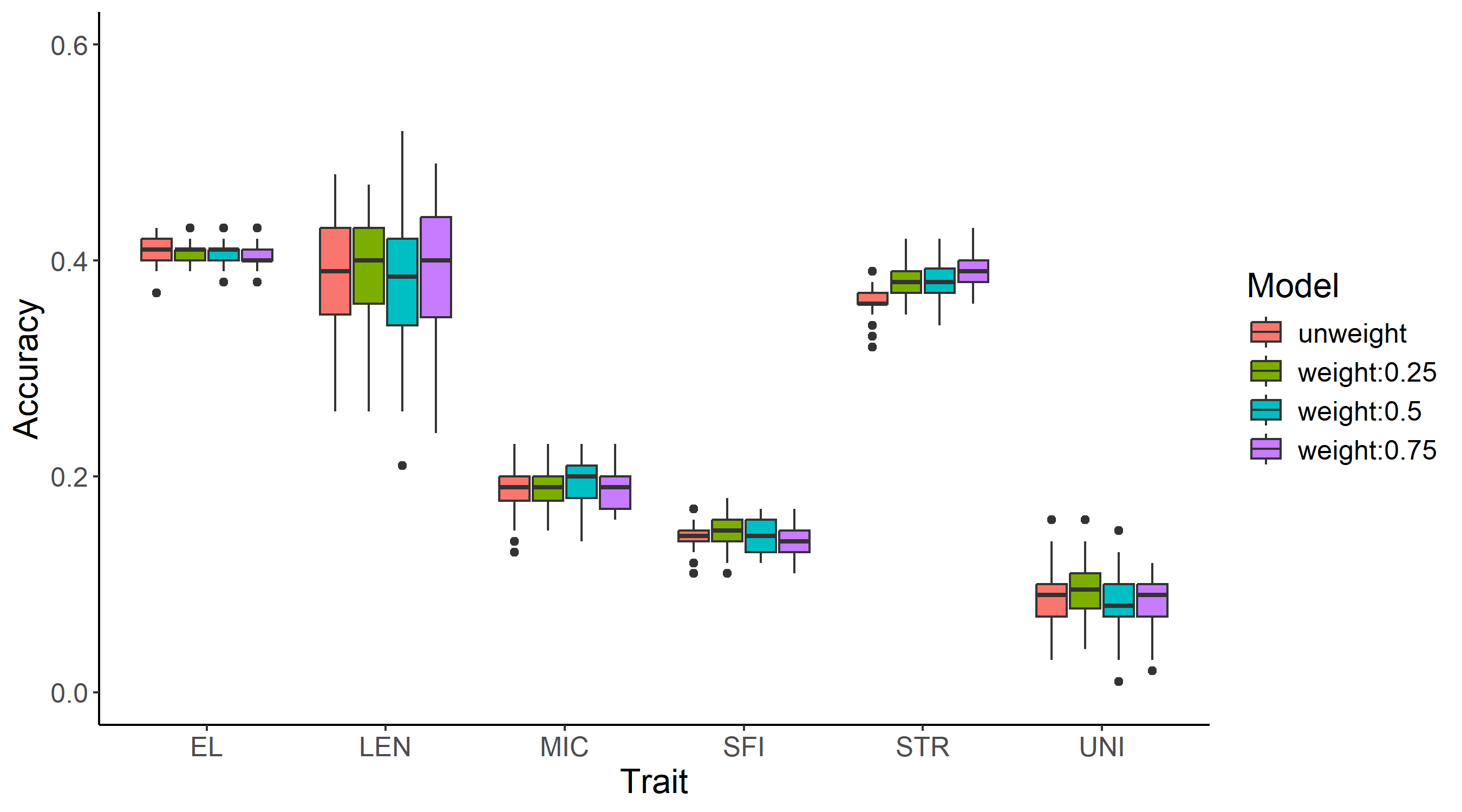


FLA11 (2^nd^ neighbour)


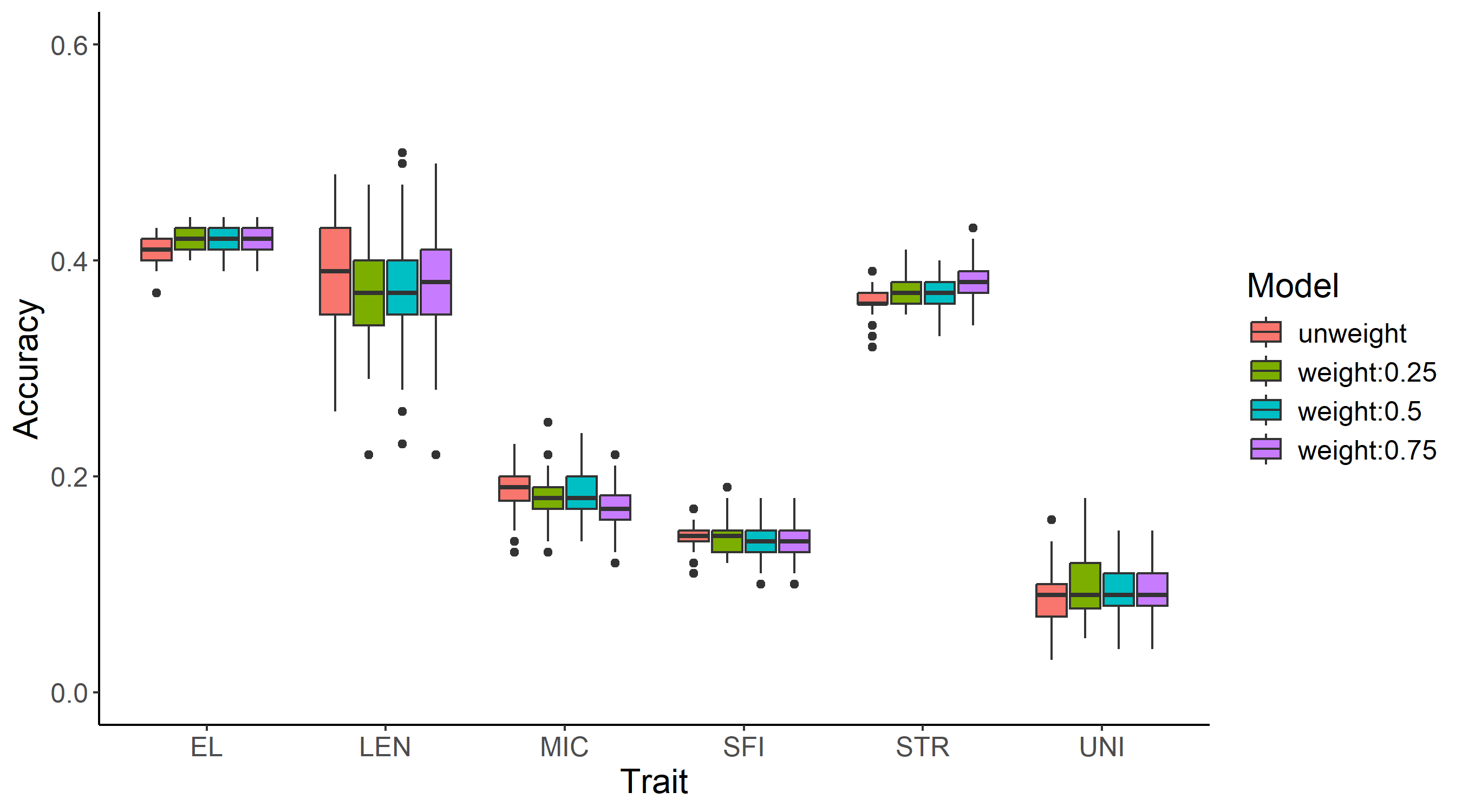


1. FLA11 (3^rd^ neighbour)


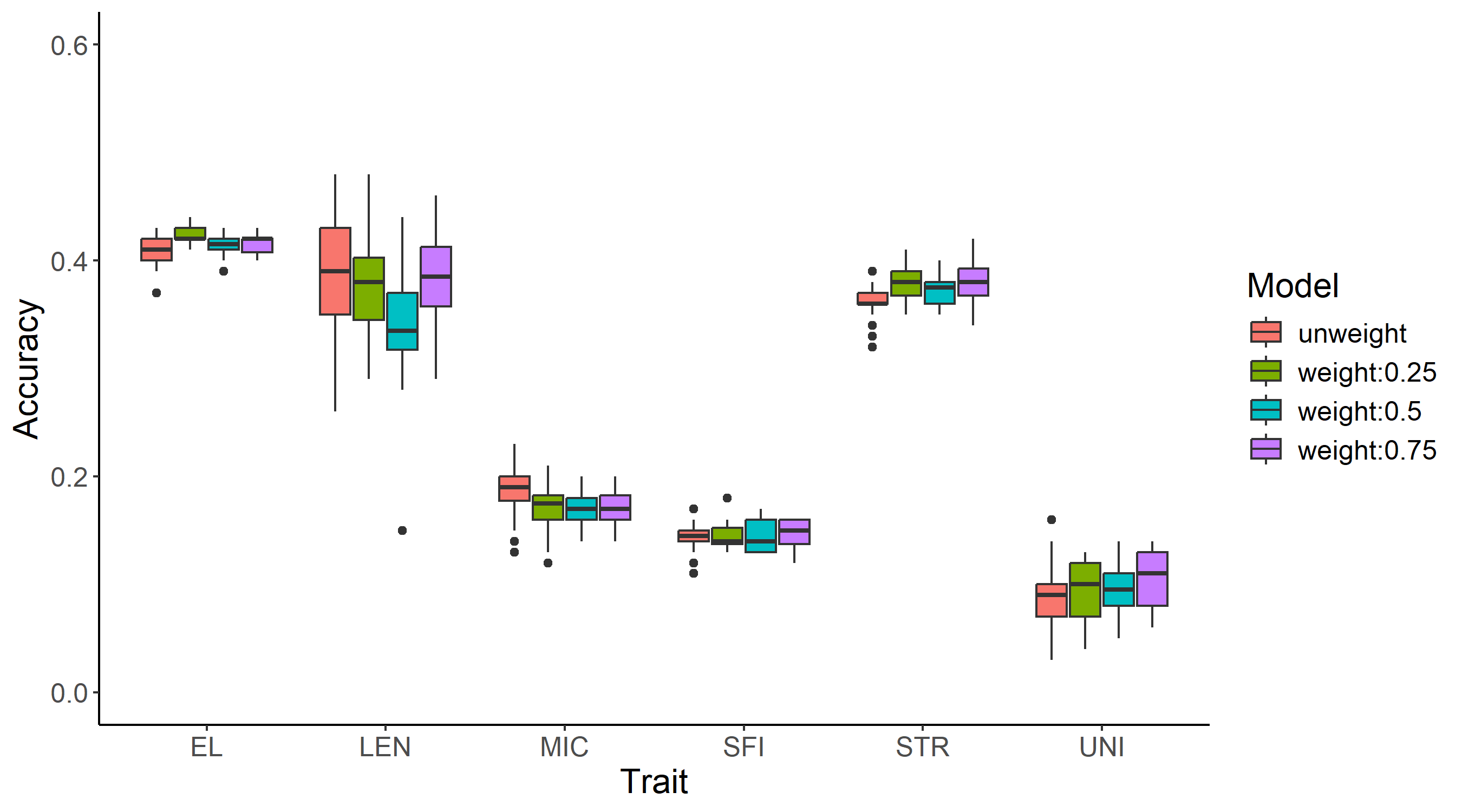


FLA12 (1^st^ neighbour)


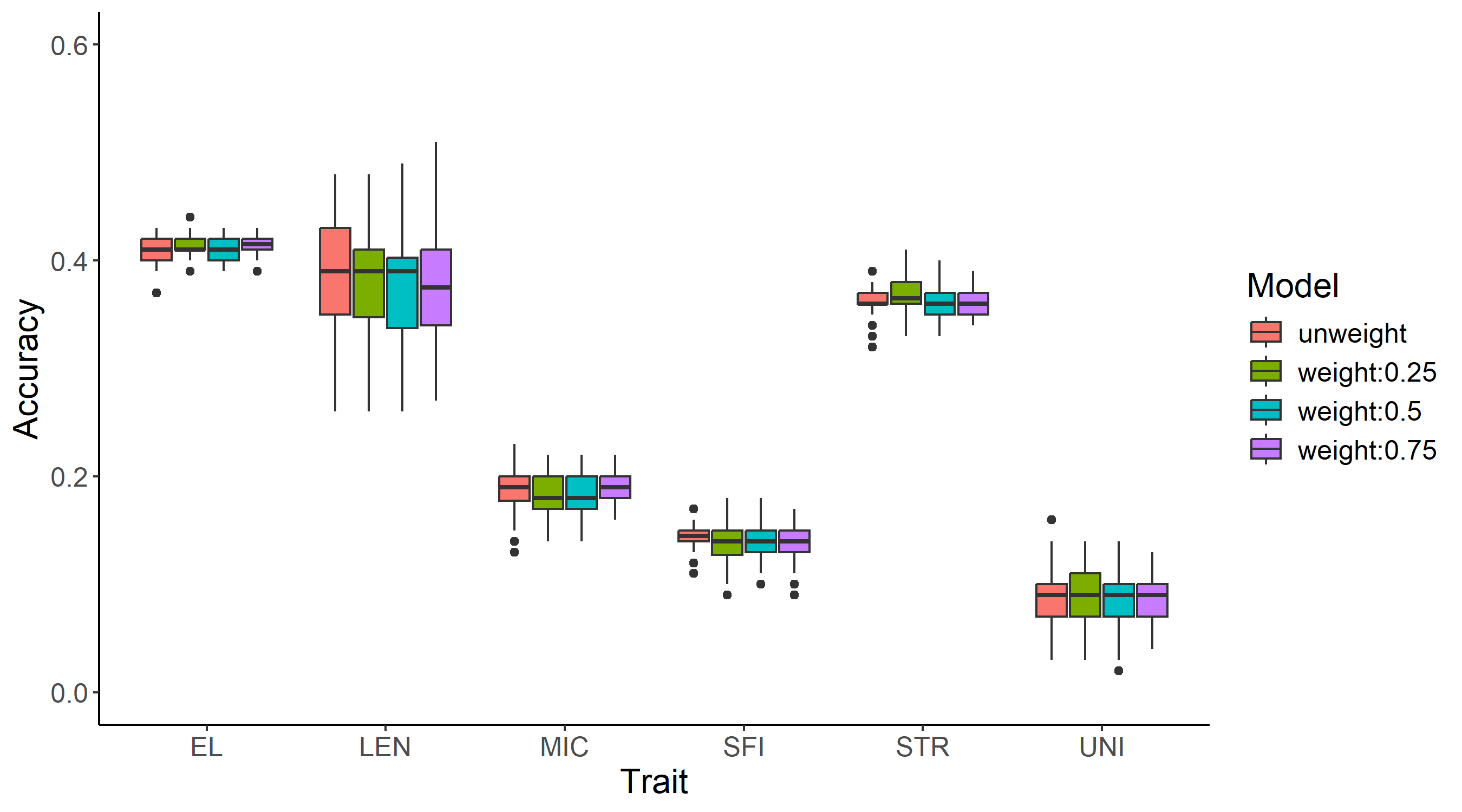


1. FLA12 (2^nd^ neighbour)


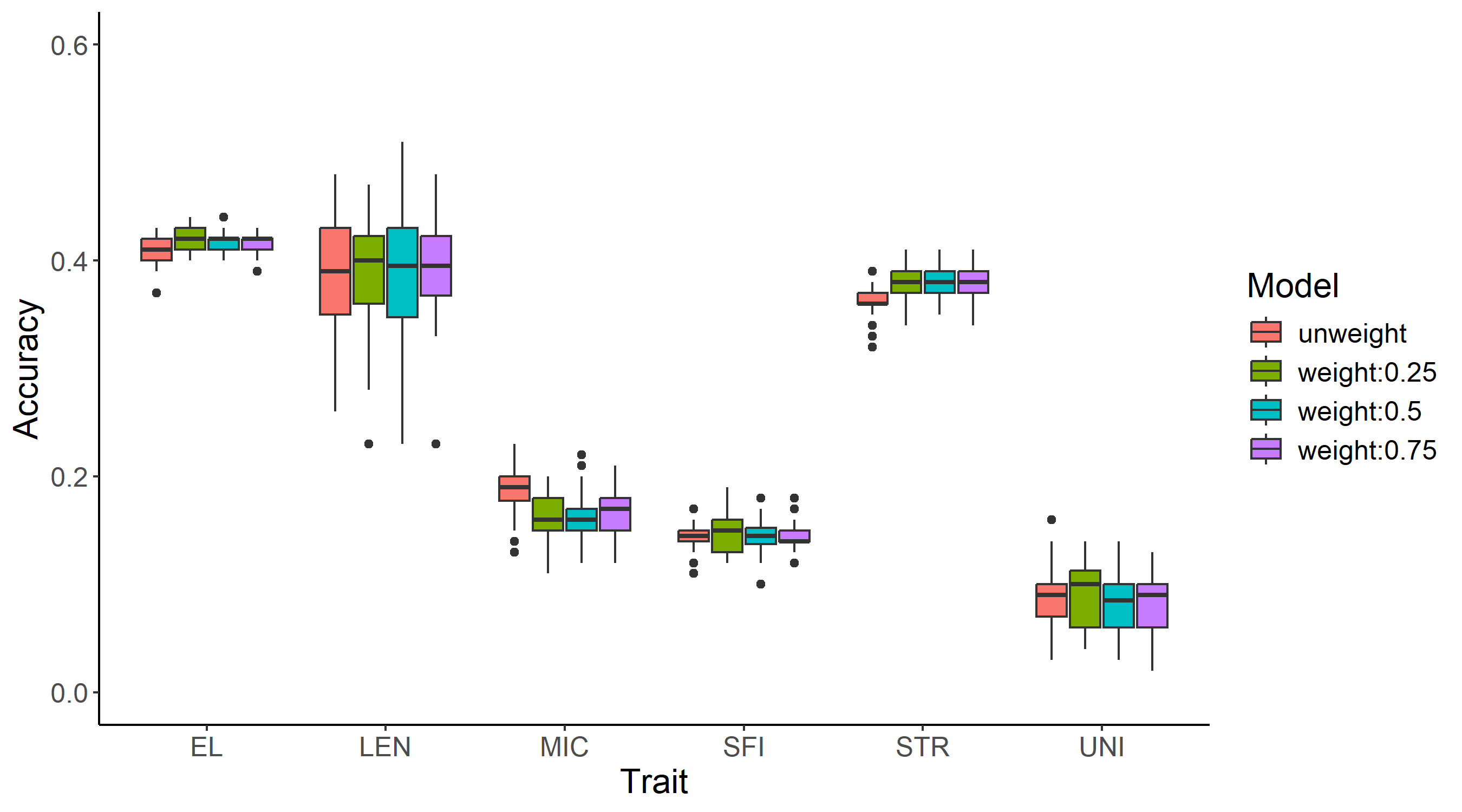


FLA12 (3^rd^ neighbour)


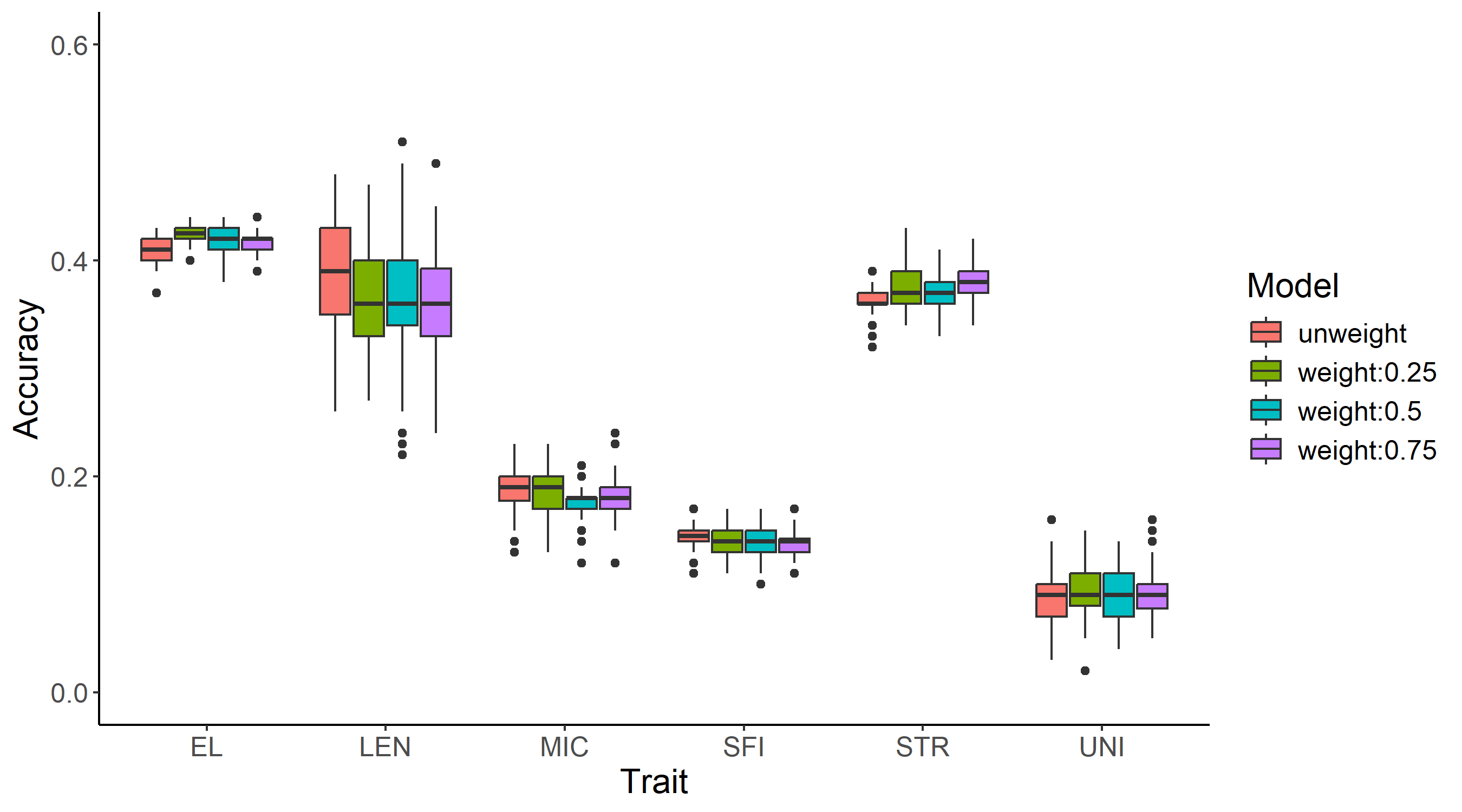

Supplement: Supplementary File 6 — GP of all 6 fibre quality traits with FLA7, FLA11, and FLA12 GCN-clusters, at the 1st, 2nd, and 3rd network neighbour level in each cluster. [file Table6.docx]
